# α-Enolase and γ-Enolase Expression in Enriched S- and N-Type SH-SY5Y Cells: Regulatory Role of Cathepsin X

**DOI:** 10.1007/s12035-025-04898-2

**Published:** 2025-04-03

**Authors:** Selena Horvat, Nace Zidar, Janko Kos, Anja Pišlar

**Affiliations:** 1 Department of Pharmaceutical Biology, Faculty of Pharmacy, University of Ljubljana, Aškerčeva 7, 1000 Ljubljana, Slovenia; 2 Department of Pharmaceutical Chemistry, Faculty of Pharmacy, University of Ljubljana, Aškerčeva 7, 1000 Ljubljana, Slovenia; 3 Department of Biotechnology, Jožef Stefan Institute, Jamova 39, 1000 Ljubljana, Slovenia

**Keywords:** Neuron-like cells, Neuronal differentiation, SH-SY5Y cell phenotypes, Enolase isoforms, Cathepsin X inhibition

## Abstract

**Abstract:**

Enolase is well-known for its role in glycolysis but also plays other roles in the central nervous system, including neuronal survival, differentiation, and axonal regeneration. Here, we investigated α- and γ-enolase expression patterns and their association with cathepsin X in distinct SH-SY5Y cell phenotypes. Enriched substrate-adherent S-type cells are characterized by large, flat morphology with extensive cytoplasm and higher expression of vimentin, while neuroblastic N-type are recognized by neurite extensions and higher expression of B-cell lymphoma 2 (Bcl-2) and growth-associated protein-43. We demonstrated that γ-enolase expression was specific to N-type cells, whereas α-enolase expression was not phenotype-specific. Moreover, a shift from ubiquitously expressed α-enolase to neuron-specific γ-enolase was observed during the enrichment and differentiation. Additionally, cathepsin X exhibited higher proteolytic activity in S-type cells. Inhibition of cathepsin X with AMS36 promoted differentiated cell morphology and increased expression of the active form of γ-enolase. Furthermore, AMS36 altered the expression of vimentin and Bcl-2, indicating a regulatory role in neuronal differentiation. Furthermore, AMS36 activated extracellular signal-regulated kinase 1/2 in N-type cells and enhanced the association between γ-enolase and tyrosine receptor kinase in both, suggesting a link between cathepsin X/γ-enolase and the key signaling pathways of differentiation. Our findings underscore the multifaceted role of enolase isoforms in SH-SY5Y cell differentiation, with α-enolase and γ-enolase showing distinct expression patterns in S- and N-type cells. The expression and activity of cathepsin X in S-type cells, along with its regulatory impact on γ-enolase in N-type cells, highlight the importance of these proteins in neuronal differentiation.

**Graphical Abstract:**

**The roles of α-enolase, γ-enolase, and cathepsin X in enriched and differentiated SH-SY5Y cell populations.** The enrichment and differentiation of SH-SY5Y cells resulted in two distinct cell phenotypes: S-type and N-type cells. S-type cells were characterized by an epithelial-like morphology, the presence of vimentin, lower γ-enolase expression, and higher cathepsin X expression. N-type cells were characterized by a neuron-like morphology, GAP-43 and Bcl-2 expression, higher γ-enolase expression, and lower cathepsin X expression. Both phenotypes expressed α-enolase. The cathepsin X inhibitor AMS36 promoted SH-SY5Y cell differentiation and enrichment into S- and N-type cells. AMS36-treated S-type cells exhibited decreased vimentin levels and increased active γ-enolase levels, indicating enhanced differentiation. AMS36-treated N-type cells exhibited decreased Bcl-2 levels, indicating further differentiation. These results highlight the differential protein expression and activity between S- and N-type cells. Furthermore, they highlight the modulatory effects of AMS36, emphasizing its potential role in promoting differentiation and altering protein expression profiles.

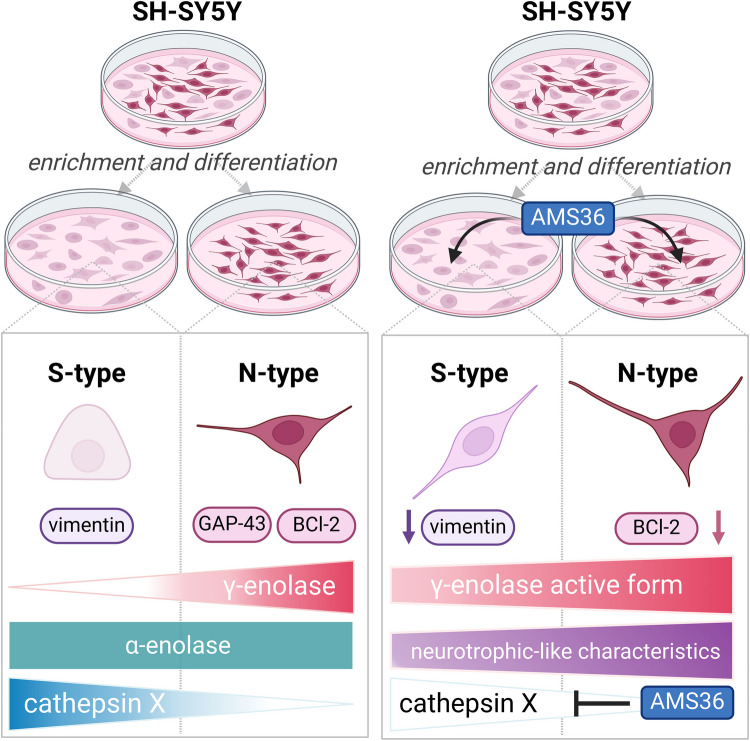

**Supplementary Information:**

The online version contains supplementary material available at 10.1007/s12035-025-04898-2.

## Introduction

Understanding the subcellular and molecular changes that occur during neuronal differentiation is crucial for identifying therapeutic targets for neurological diseases. The SH-SY5Y neuroblastoma cell line serves as a versatile research model for studying these changes, displaying three distinct phenotypes: neuroblast-like N-type cells, substrate-adherent epithelial-like S-type cells, and intermediate I-type cells [1–3]. This diversity within the SH-SY5Y cell line makes it an invaluable tool for exploring the various aspects of cell differentiation [4]. Each phenotype can be distinguished by specific molecular markers and exhibits unique morphological and tumorigenic behavior in vitro [5].

N-type cells are the most prevalent SH-SY5Y cells [6, 7]. These cells are characterized by their small, round cell bodies and short neurite-like processes and express mature neuronal markers [3, 6, 7]. S-type cells are multipotent progenitors of Schwann cells, melanocytes, and glial cells, thus representing the non-neuronal derivatives of the neural crest [3]. These cells are characterized by their expansive, flat cellular bodies, abundant cytoplasm, lacking neuritic outgrowths, and strong substrate adherence in vitro [6, 7]. Unlike N-type cells, S-type cells do not possess catecholaminergic enzymatic markers, indicating their specialized roles in cellular processes such as adhesion, migration, and interactions with the extracellular matrix [5]*.* I-type cells exhibit characteristics of both N- and S-type cells and are recognized as a progenitor population, capable of self-renewal and bidirectional differentiation [1]. Moreover, retinoic acid suppresses SH-SY5Y cell proliferation and triggers differentiation, thereby enabling the selection and enrichment of either N- or S-phenotypes based on their distinct substrate adherence properties [4]. This capacity for phenotype-specific differentiation enhances the versatility and applicability of the SH-SY5Y cell line for focused investigations of differentiation pathways, signaling mechanisms, and potential drug responses.

The glycolytic enzyme γ-enolase is a well-known neuronal marker with a less known role in neuronal differentiation [8]. In mammalian brains, enolase exists as two specific isoenzymes: γ-enolase (also known as neuron-specific enolase; γγ and αγ) and α-enolase (also known as non-neuronal enolase; αα). α-Enolase is ubiquitously expressed but is replaced by γ-enolase isoforms during neuronal development, indicating a functional shift towards the specialized roles of γ-enolase in mature neurons [9–11]. γ-Enolase, specifically its C-terminal part, shares many characteristics with neurotrophins and has been characterized as a neurotrophic-like factor with substantial effects on neuronal growth, differentiation, survival, and regeneration [12, 13]. Moreover, γ-enolase plays a pivotal role in enhancing neuronal survival and differentiation through the activation of key intracellular signaling pathways, namely phosphatidylinositol 3-kinase and mitogen-activated protein kinase [12]. The neurotrophic activity of γ-enolase is regulated by the lysosomal peptidase cathepsin X, which cleaves the C-terminal dipeptide of γ-enolase, thereby abolishing its neurotrophic activity and regulating neuritogenesis and the survival of neuron-like cells [14–16].

However, the role of γ-enolase in cell survival and neurite growth among the distinct SH-SY5Y phenotypes is still unknown. Considering the phenotypic diversity and differentiation potential of the SH-SY5Y cell line, we hypothesize that γ-enolase expression is phenotype-specific in SH-SY5Y cells and undergoes significant changes during differentiation, characterized by a transition from the ubiquitously expressed α-enolase to the neuron-specific γ-enolase in neuronal-like cells. In this study, we enriched and differentiated the SH-SY5Y cell line into S- and N-type cells, in which we characterized the distinct expression patterns of enolase isoforms. Our results revealed that the expression of γ-enolase was increased in N-type SH-SY5Y cells. During enrichment and differentiation, a shift from ubiquitously expressed α-enolase to neuron-specific γ-enolase occurred. Additionally, we determined the regulatory role of cathepsin X on the expression of the active form of γ-enolase and its subsequent effects on cell differentiation.

## Materials and Methods

### Reagents

Retinoic acid (Sigma-Aldrich, St. Louis, MO, USA) was prepared as a 10 mM stock solution in dimethyl sulfoxide (DMSO; Sigma-Aldrich). The irreversible inhibitor of cathepsin X, AMS36, was synthesized as reported [17] and prepared as a 10 mM stock solution in DMSO (Sigma-Aldrich).

### Cell Culture

Human neuroblastoma SH-SY5Y cells were obtained from the American Type Culture Collection (CRL-2266; Manassas, VA, USA). Cells were cultured in Dulbecco’s modified Eagle’s medium (DMEM)/F12 (Sigma-Aldrich) supplemented with 10% (v/v) fetal bovine serum (Thermo Fisher Scientific, Waltham, MA, USA) and 1% penicillin–streptomycin (Sigma-Aldrich). Cells were maintained at 37 °C in a humidified atmosphere containing 5% CO_2_ and grown to 80% confluence.

### Enrichment of S- and N-Type Cells and Treatment

To enrich S- and N-type cell populations, we adapted the protocol from Bell et al. [6]. This protocol (Supplementary Fig. S1) involves removing less adherent N-type cells and thus promoting S-type enrichment. Cells were seeded onto six-well plates at a density of 1 × 10^5^ cells/mL for control groups and 3 × 10^5^ cells/mL for enrichment groups and cultured in growth media for at least 24 h before starting enrichment. The next day, enrichment group wells were washed with pre-warmed Dulbecco’s phosphate-buffered saline (DPBS; Sigma-Aldrich) to remove loosely attached cells. These cells were then transferred to a new culture plate to specifically cultivate N-type cells. Cells that remained adherent in the original wells were identified as S-type cells. All cells, including control and enriched cells, were then cultured in differentiation media containing DMEM/F12 supplemented with 2% (v/v) fetal bovine serum and 0.25% penicillin–streptomycin. After 4 and 7 days, retinoic acid at a final concentration of 5 µM was added to the reduced serum media to support the differentiation process of control, S-type, and N-type cells. Control cells were treated with an equal volume of vehicle of retinoic acid, DMSO. Cells were enriched for 10 days, and cell morphology was assessed on days 4, 7, and 10 of the enrichment process (Supplementary Fig. S1).

For cell treatment, AMS36 at a final concentration of 10 μM was added to inhibit cathepsin X after 4 and 7 days in differentiation media. Non-treated control cells were treated with an equal volume of vehicle (DMSO).

### Evaluation of Neurite Length

Cells were seeded (at 1 × 10^5^ or 3 × 10^5^ cells/mL) in growth media in six-well culture plates in duplicates. The next day, cells were enriched (as described above) and subjected to differentiation for 10 days. Neurite length was evaluated by morphological examination and captured using the EVOS Cell Imaging System (Thermo Fisher Scientific). ImageJ software was used to determine the length of neurites (in pixels) that were longer than the corresponding cell diameter. Approximately 100 cells per condition were measured in each experiment. Three independent experiments (*N* = 3) were performed, each in duplicate. Data are expressed relative to control, i.e., non-differentiated cells, and as means ± SEM (one-way analysis of variance (ANOVA), Tukey’s test; * *P* < 0.05).

### Cell Proliferation Assay

Cells were seeded (at 1 × 10^5^ or 3 × 10^5^ cells/mL) in growth media in six-well culture plates in duplicates. The next day, cells were stained with CellTrace carboxyfluorescein succinimidyl ester (CFSE) reagent (Thermo Fisher Scientific) at a concentration of 1 μM according to the manufacturer’s protocol. Subsequently, the cells were enriched (as described above) and subjected to differentiation for 4, 7, and 10 days. Then, cells were collected, and their mean fluorescence intensities were measured in the BL-1 channel using a flow cytometer (Attune NxT, Thermo Fisher Scientific). The obtained data were analyzed in FlowJo software (Tree star Inc., Ashland, OR, USA), and mean CFSE fluorescence intensities were normalized to the control cells. Three independent experiments with two replicates per differentiation condition were performed. Three independent experiments (*N* = 3) were performed, each in duplicate. Data are expressed relative to control and as means ± SEM (one-way ANOVA, Tukey’s test; * *P* < 0.05).

### Cell Lysate Preparation

After 4, 7, or 10 days of cell enrichment and differentiation, cells were scraped off wells, washed with phosphate buffer saline, pH 7.4 (PBS), and lysed with either of two lysis buffers: for assessing protein expression via western blotting and enzyme-linked immunosorbent assay (ELISA) (50 mM HEPES, pH 6.5, 150 mM NaCl, 1 mM EDTA, 0.25% Triton X-100) or for quantifying cathepsin X activity (50 mM Na-acetate, pH 5.5, 1 mM EDTA, 100 mM NaCl, 0.25% Triton X-100). After lysis, they were incubated for 30 min on ice. To evaluate the expression of both phosphorylated and total extracellular signal-regulated kinase (ERK1/2) and to perform immunoprecipitation, lysates were prepared in HEPES lysis buffer supplemented with a cocktail of protease and phosphatase inhibitors (Thermo Fisher Scientific) and incubated for 30 min on ice. Whole-cell lysates were preserved at − 80 °C and subjected to freeze–thaw cycles. Cells were sonicated and centrifuged at 15,000 × g for 15 min at 4 °C. The protein concentrations in the obtained lysates were quantified using the DC Protein Assay (Bio-Rad, Hercules, CA, USA). Bovine serum albumin (BSA; Sigma-Aldrich) was used as a protein standard to determine protein concentrations.

### Immunoprecipitation and Western Blotting

For immunoprecipitation, the protein concentrations were adjusted by adding lysis buffer, pH 8.0, to a final concentration of 100 μg in 50 μL. Then 25 µg/mL of goat polyclonal anti-cathepsin X (AF934; R&D Systems, Minneapolis, MN, USA) specific antibody or normal goat IgG control antibody (AB-108-C; R&D Systems) was added and incubated overnight at 4 °C. To purify the immune complexes, 50 μL of this mixture was added to washed protein A-Sepharose beads (ab193256; Abcam, Cambridge, UK) and mixed for additional 2 h at 4 °C. The beads were then washed with binding buffer (0.14 M phosphate buffer), pH 8.2, and the immune complexes eluted by boiling in SDS-PAGE buffer.

For western blot, equal protein concentrations from whole-cell lysates (1 μg/μL) were denatured by the addition of SDS-PAGE buffer, heated for 5 min, and resolved by SDS-PAGE on 12% Tris–glycine gels. The proteins were then transferred to polyvinylidene difluoride membranes using iBlot (Thermo Fisher Scientific). Membranes were blocked in 5% (w/v) non-fat dried milk powder in Tris-buffered saline with Tween 20 (TBST; 20 mM Tris/HCl, pH 7.4, 137 mM NaCl, and 0.1% Tween 20) at room temperature for 1 h. Subsequently, the membranes were incubated overnight at 4 °C with primary antibodies in TBST containing 3% (w/v) BSA. The following primary antibodies and dilutions were used: mouse monoclonal anti-vimentin (1:500; sc-6260), mouse monoclonal anti-neurofilament light chain protein (1:250; sc-20012), mouse monoclonal anti-tyrosine hydroxylase (1:500; sc-25269), mouse monoclonal anti-α-enolase (1:500; sc-100812), mouse monoclonal anti-γ-enolase raised against amino acids 416–433 of γ-enolase, determining the active form of γ-enolase (1:250; sc-21738), mouse monoclonal anti-γ-enolase raised against amino acids 271–285 of γ-enolase, determining the total form of γ-enolase (1:350; sc-21737), rabbit polyclonal anti-ERK1 (1:1000; sc-93), and rabbit polyclonal anti-ERK2 (1:2500; sc-154; all from Santa Cruz Biotechnology, Dallas, TX, USA); rabbit monoclonal anti-B-cell lymphoma 2 (Bcl-2) (1:1000; 4223) and rabbit polyclonal anti-phospho ERK1/2 (Thr202/Try204) (1:1000; 9101S; both from Cell Signaling Technology, Danvers, MA, USA); rabbit monoclonal anti-growth-associated protein-43 (GAP-43) (1:1000; ab75810; Abcam); rabbit polyclonal anti-GAPDH (1:10,000; 10,494–1-AP; Proteintech, Rosemont, IL, USA); and goat polyclonal anti-cathepsin X (1:500; AF934; R&D Systems). The membrane for goat anti-cathepsin X antibody incubation was blocked with 1.5% (w/v) non-fat dried milk powder in TBST and diluted in TBST containing 1.5% non-fat dried milk powder and 1% (w/v) BSA.

After washing, the following secondary horseradish-peroxidase-conjugated antibodies in TBST containing 5% (w/v) non-fat dried milk powder were added for 1 h: goat polyclonal anti-mouse (1:5000; 111–035–068), goat polyclonal anti-rabbit (1:5000; 111–035–045; both from Jackson ImmunoResearch, West Grove, PA, USA), and mouse monoclonal anti-goat (1:2000; sc-2354; Santa Cruz Biotechnology) antibodies. After washing, protein bands were visualized with enhanced chemiluminescence detection kits (Thermo Fisher Scientific) and recorded with a G:Box imager (Syngene, UK). When necessary, membranes were stripped with stripping buffer (62.5 mM Tris/HCl, pH 5.7, 100 mM 2-mercaptoethanol, and 2% SDS) for 1 h at 65 °C. Band intensities were quantified using Gene Tools software (Sygene). To determine the protein levels, values were expressed as a ratio to GAPDH and were normalized to the control sample. At least two (*N* = 2) independent experiments were performed. Data are expressed relative to control and as means ± SEM (one-way ANOVA, Tukey’s test; * *P* < 0.05).

### ELISA

The protein levels of α-enolase and the active and total forms of γ-enolase in SH-SY5Y cells were determined by ELISA. To measure the active and total forms of γ-enolase, microtiter plates were coated with equal aliquots of mouse monoclonal anti-enolase antibody (1:20, Santa Cruz Biotechnology) in 0.01 M carbonate/bicarbonate buffer, pH 9.6, and incubated at 4 °C overnight. After incubation with blocking buffer (2% BSA in PBS, pH 7.4), for 1 h at room temperature, samples containing equal protein amounts (25 µg) were added. Following a 2 h incubation at 37 °C, the wells were washed and filled with mouse monoclonal anti-enolase antibody conjugated with horseradish peroxidase (1:50; sc-271384; Santa Cruz Biotechnology) in a blocking buffer. After an additional 1.5 h incubation at 37 °C, 100 µL of 3,3′,5,5′-tetramethylbenzidine substrate (Sigma-Aldrich) was added to each well. The reaction was stopped after 15 min by adding 50 µL of 2 M H_2_SO_4_ to each well. Protein levels were determined by measuring the absorbance at 450 nm using a Tecan Safire spectrophotometer (Tecan Safire^2^, Switzerland), and protein levels were expressed relative to the control. Three independent experiments (*N* = 3) were performed, each in duplicate. Data are expressed relative to control and as means ± SEM (one-way ANOVA, Tukey’s test; * *P* < 0.05).

Cathepsin X protein levels were determined with the Human Cathepsin Z ELISA Kit (2,023,308; MyBioSource, San Diego, CA, USA) following the manufacturer’s instructions. Briefly, standards and samples with equal protein amounts (50 μg) were added to wells and incubated for 1 h at 37 °C. Then, Detection Reagent A was added to wells and incubated for 1 h at 37 °C, after which the wells were washed and filled with Detection Reagent B. After 30 min of incubation at 37 °C, Substrate Solution was added. After 20 min of incubation at 37 °C, the reaction was terminated by adding Stop Solution. The protein levels were determined by measuring the absorbance at 450 nm using a microplate reader (Tecan Safire^2^), and protein levels were expressed relative to the control. Three independent experiments (*N* = 3) were performed, each in duplicate. Data are expressed relative to control and as means ± SEM (one-way ANOVA, Tukey’s test; * *P* < 0.05).

### Double Immunofluorescence Staining

Cells were cultured on glass coverslips at a concentration of 2 × 10^4^ cells/mL and underwent a 10-day enrichment and differentiation protocol. After 10 days, cells were fixed in 4% (w/v) paraformaldehyde (Electron Microscopy Sciences, Hatfield, PA) in PBS for 30 min, washed with PBS, and permeabilized with 0.5% Tween-20 (Sigma-Aldrich) in PBS (pH 7.4) for 10 min. Non-specific staining was blocked with 10% normal donkey serum (Sigma-Aldrich) in PBS containing 0.05% Triton X-100 (Sigma-Aldrich) for 30 min at room temperature. Cells were then incubated with either rabbit polyclonal anti-α-enolase (1:200, 11,204–1-AP, Proteintech) or mouse monoclonal anti-γ-enolase (1:50, 66,150–1-Ig; Proteintech) and goat polyclonal anti-cathepsin X (1:75, AF934; R&D Systems) or rabbit monoclonal anti-Trk (1:200, 92,991; Cell Signaling Technology) antibodies in blocking solution for 2 h at room temperature. Afterwards, cells were washed with PBS and further incubated with Alexa Fluor 488- and Alexa Fluor 555-labelled secondary antibodies (1:1000, Thermo Fisher Scientific) for an additional 1.5 h. After washing with PBS, ProLong Gold antifade reagent with DAPI (Thermo Fisher Scientific) was used to mount the coverslips onto glass slides. Fluorescence microscopy was performed using a confocal microscope (LSM 710; Carl Zeiss, Oberkochen, Germany) with the ZEN 3.4 image software. Co-localization areas were analyzed for ≥ 10 cells, and their quantification was represented by the mean number of pixels in the third quadrant in the scatter plots for two fluorescence intensities. At least two (*N* = 2) independent experiments were performed. Data are expressed relative to control and as means ± SEM (one-way ANOVA, Tukey’s test; * *P* < 0.05).

### Cathepsin X Activity Assay

To evaluate cathepsin X activity, cell lysates were diluted in buffer (50 mM acetate, pH 5.5, 5 mM dithiothreitol (Sigma-Aldrich), and 1.5 mM EDTA (Sigma-Aldrich)) to a protein concentration of 0.125 mg/mL and incubated at 37 °C for 10 min. In duplicate, 95 µL of lysates was transferred into the wells of a black microtiter plate (Nunclon Delta Surface; Thermo Fisher Scientific) containing 5 µL of the previously added fluorogenic substrate Abz-FEK(Dnp)-OH (Puzer et al., 2005) at a final concentration of 5.9 µM. The subsequent degradation was continuously monitored at 320 ± 5 nm excitation and 420 ± 5 nm emission using a Tecan Safire spectrophotometer (Tecan Safire^2^). Activity measurements were analyzed with Magellan™ 7.2 SP1 software (Tecan Safire^2^), and the resulting data, expressed in relative fluorescence units, were normalized to the control sample. Three independent experiments (*N* = 3) were performed, each in duplicate. Data are expressed as means ± SEM (one-way ANOVA, Tukey’s test; * *P* < 0.05).

### Enolase Activity Assay

Enolase assays were performed according to the Enolase Assay Kit instructions (ab241024; Abcam). Briefly, cell lysates were prepared using the Enolase buffer, and protein concentrations were determined using a method described in the previous *Cell lysate preparation* paragraph. Initially, standards and samples were prepared (1 µg/mL) and loaded in duplicate onto the plate, forming two separate sets of samples. Additionally, 100-fold and 1000-fold dilutions of the Positive Control were prepared and loaded onto the plate in duplicate. The reaction and a corresponding background control mixture (substituting substrate with buffer) were formulated and applied to the plate. Absorbance was measured at 570 nm using a Tecan Safire spectrophotometer (Tecan Safire^2^) in kinetic mode for 60 min at room temperature. Sample absorbances were corrected using their respective background controls. Results were expressed as the slope of the linear portion of the reaction curve over time, with data normalized to control cells. Two independent experiments (*N* = 2) were performed, each in duplicate. Data are expressed relative to control and as means ± SEM (one-way ANOVA, Tukey’s test; * *P* < 0.05).

### Statistical Analysis

The results were obtained from at least two independent experiments, each performed in at least duplicate, and are shown as mean ± standard error of the mean (SEM). Statistical analysis was performed with the two-tailed *t*-test to compare two groups or one-way analysis of variance to compare three or more groups followed by Tukey’s post hoc test using GraphPad Prism, version 10. *P* < 0.05 was considered statistically significant.

## Results

### Enriched S- and N-Type Cells Showed Distinct Phenotypes

**Fig. 1:**
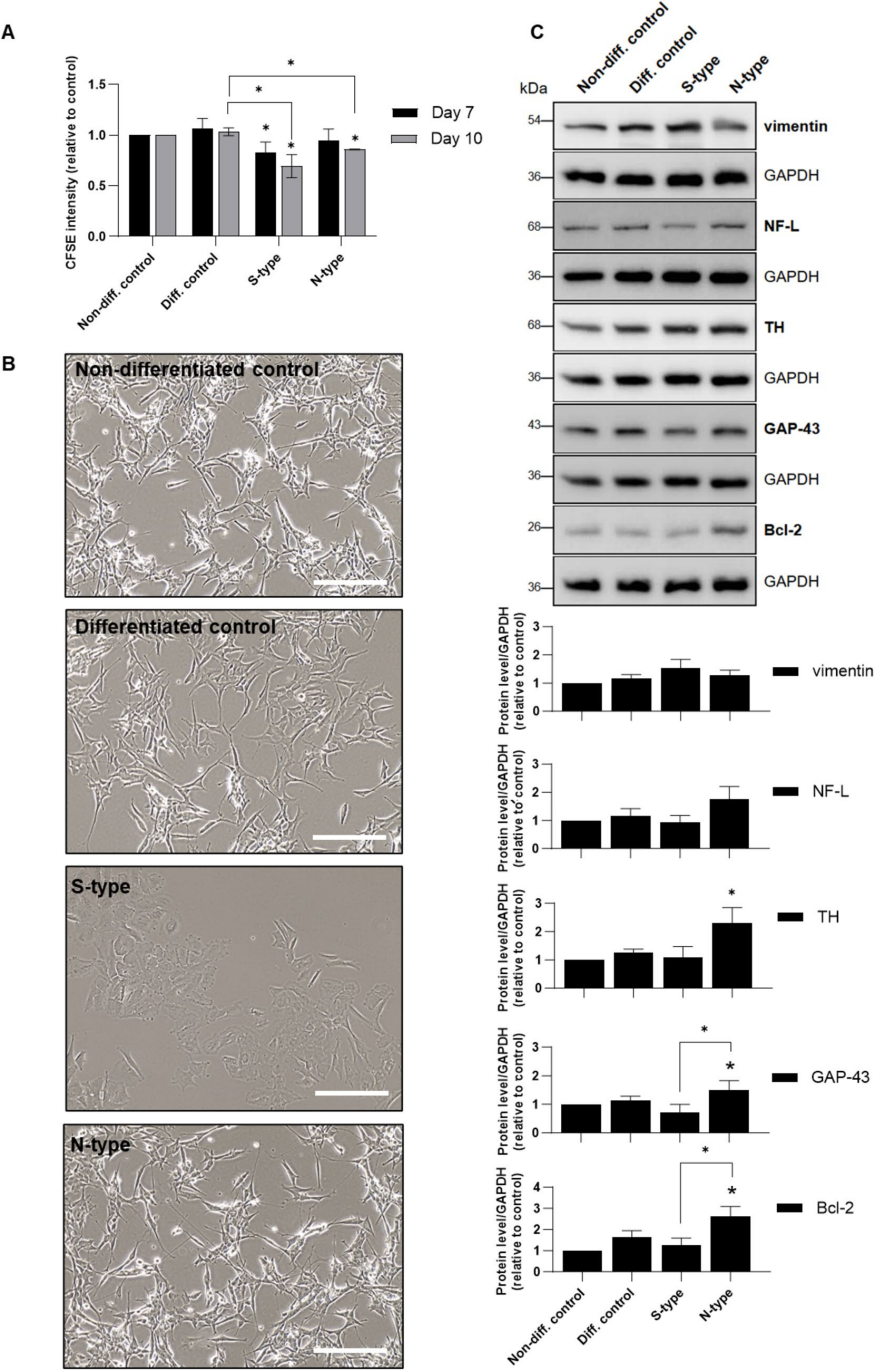
The effects of the enrichment and differentiation protocol on cell proliferation, morphology, and expression of specific markers in SH-SY5Y cell populations. **A** Proliferation rates of SH-SY5Y cells enriched and differentiated for 7 and 10 days were assessed with carboxyfluorescein succinimidyl ester (CFSE) and flow cytometry. Three independent experiments (*N* = 3) were performed, each in duplicate. **B** Representative phase-contrast images of SH-SY5Y cell populations on day 10 of the enrichment and differentiation protocol. Scale bars: 100 μm. **C** Representative western blots (top) and quantification (bottom) of the expression of vimentin, neurofilament light chain protein (NF-L), tyrosine hydroxylase (TH), growth-associated protein-43 (GAP-43), and B-cell lymphoma 2 (Bcl-2) after 10 days of enrichment and differentiation. Protein levels are normalized to GAPDH. At least two independent experiments (*N* = 2) were performed. Data are expressed relative to control and as means ± SEM (one-way ANOVA, Tukey’s test; * *P* < 0.05)

To elucidate the distinct characteristics of S- and N-type SH-SY5Y cells, we first subjected cells to enrichment and differentiation and then analyzed their morphological characteristics, proliferation rates, and specific marker expressions. Cell proliferation rates were assessed on days 7 and 10. Throughout the enrichment protocol, S-type cells exhibited significantly lower CFSE levels and thus higher proliferation rates compared to control and N-type cells (Fig. 1A). After 10 days, S-type cells were morphologically characterized by large, flat cell bodies with strong substrate adherence, differentiating into a more epithelial-like phenotype. By contrast, N-type cells displayed smaller, slightly elongated cell bodies and branched neurite-like extensions differentiating towards a neuronal lineage (Fig. 1B). Western blotting of specific cell markers further defined the distinct cell phenotypes (Fig. 1C). S-type cells exhibited higher levels of vimentin, a marker of non-neuronal cells. Conversely, N-type cells exhibited higher levels of neurofilament light chain protein, a neuron-specific cytoskeletal component, and tyrosine hydroxylase, a marker for differentiated neuron-like cells, indicative of neuronal differentiation. Additionally, N-type cells showed increased levels of GAP-43 and anti-apoptotic Bcl-2, which are typically expressed in sympathetic neurons. These results demonstrate distinct morphologies and expressions of cell-specific markers, underscoring the distinct differentiation and phenotypes of S- and N-type cells. As such, they validate our enrichment and differentiation protocol.

### γ-Enolase Expression is Specific for N-type Cells

**Fig. 2:**
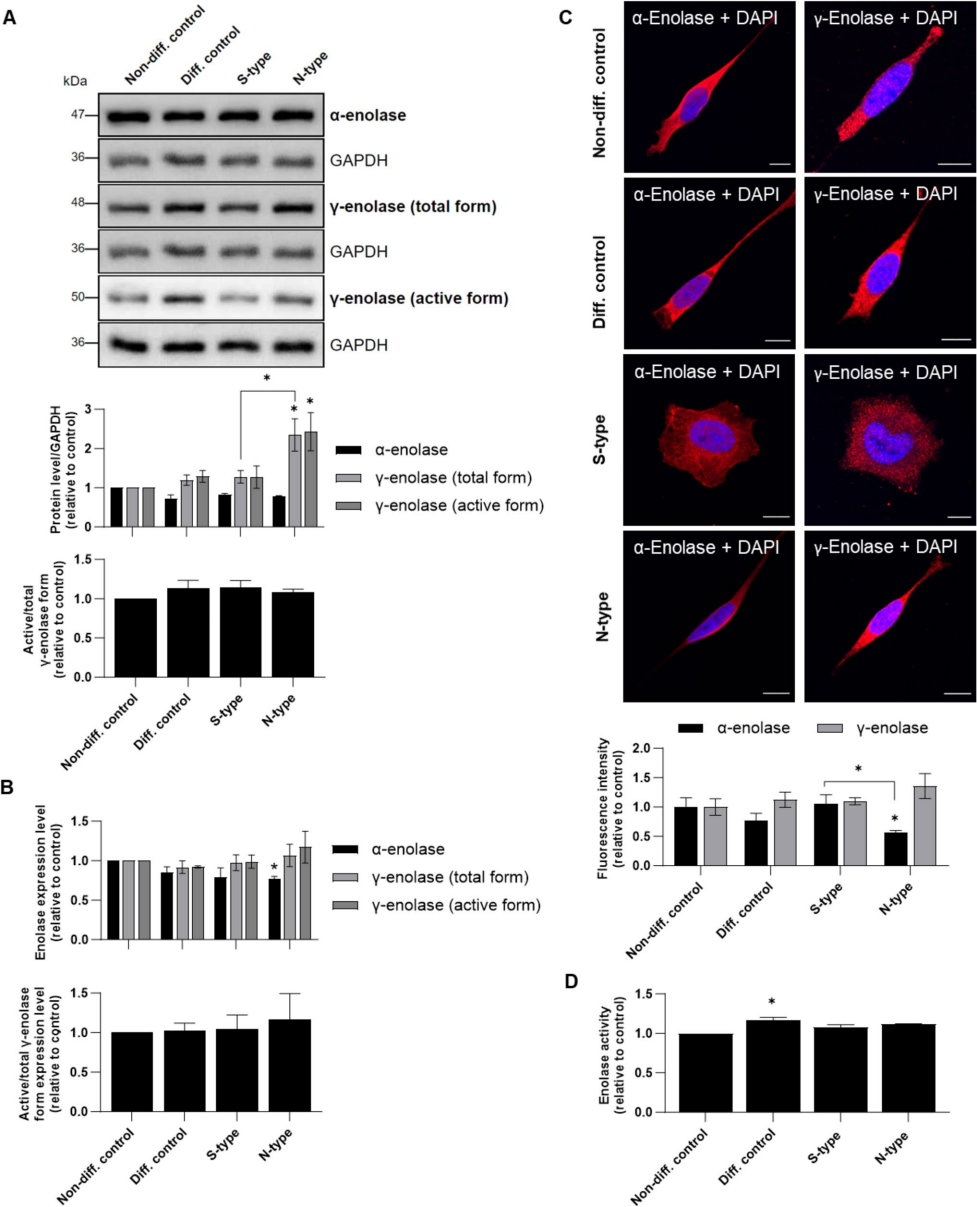
Protein expression of α-enolase and γ-enolase in enriched and differentiated SH-SY5Y cell populations. **A** Representative western blots (top) and quantification (bottom) of the expression of α-enolase, γ-enolase (total and active forms), and the ratio of active-to-total γ-enolase. Protein levels are normalized to GAPDH. At least two independent experiments (*N* = 2) were performed. (**B**) ELISA results for α-enolase and γ-enolase (total and active forms) protein expression. Three independent experiments (*N* = 3) were performed, each in duplicate. **C** Representative images of immunofluorescence staining for α-enolase (*left*) and γ-enolase (*right*). Nuclei were counterstained with DAPI (blue). The bottom graph represents quantification of the relative fluorescence intensity. Two independent experiments (*N* = 2) were performed. Scale bars: 10 μm. **D** Enolase activity assay. Two independent experiments (*N* = 2) were performed, each in duplicate. Data were obtained after 10 days of enrichment and differentiation and are expressed relative to control and as means ± SEM (one-way ANOVA, Tukey’s test; * *P* < 0.05)

To determine whether γ-enolase, a well-known neuronal marker, is differentially expressed in SH-SY5Y cell types, we assessed enolase isoform levels in SH-SY5Y cells after the 10-day enrichment and differentiation protocol. α-Enolase levels did not differ between S- and N-type cells but were slightly lower in S- and N-type cells compared to control cells. Conversely, γ-enolase levels were higher in N-type cells compared to control and S-type cells (Fig. 2A). Similar enolase isoform levels were also determined by ELISA (Fig. 2B) and confocal microscopy (Fig. 2C). The latter showed decreased and increased fluorescence intensities of α-enolase and γ-enolase in N-type cells, respectively (Fig. 2C). Interestingly, western blot and ELISA analyses revealed no significant differences between active and total γ-enolase forms across the cell phenotypes (Fig. 2A, B). Further analysis of α- and γ-enolase isoform levels revealed a progressive shift in their expression profiles (Supplemental Fig. S2A). On day 4 of enrichment, α-enolase levels were higher than γ-enolase levels in S-type cells. By day 7, this difference had diminished. By day 10, γ-enolase levels surpassed those of α-enolase, significantly so in N-type cells. Further profiling confirmed these findings, as evidenced by the differential expression of GAP-43 and Bcl-2 with time (Supplemental Fig. S2B). Furthermore, the enolase glycolytic activity assay revealed no significant differences between the cell phenotypes on day 10; however, higher glycolytic activity was observed in the differentiated control cells (Fig. 2D). These results reflect the changes in enolase isoform expression during cell enrichment and differentiation and suggest a potential role of γ-enolase in N-type cells.

### Cathepsin X Expression and Activity is Phenotype Specific

**Fig. 3:**
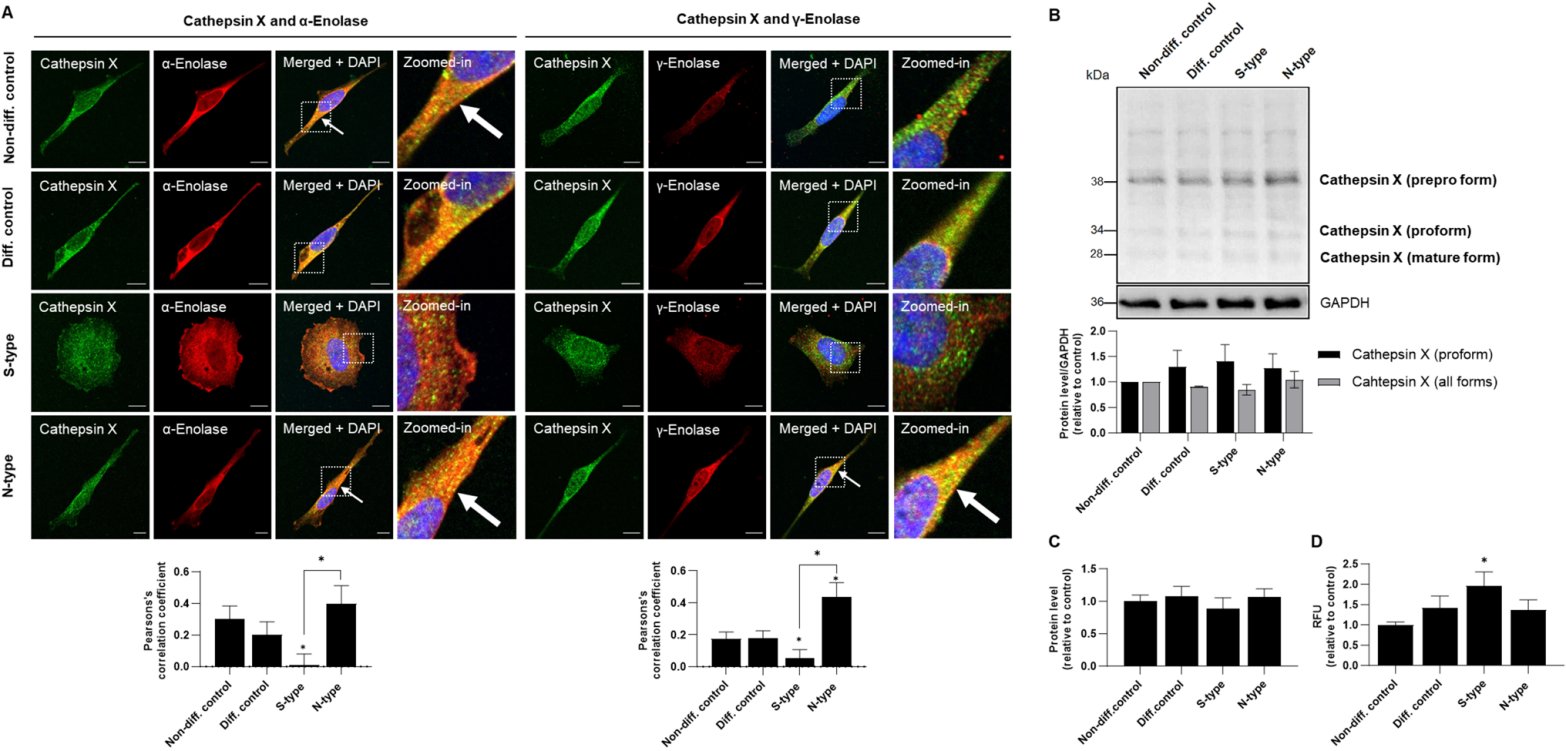
Cathepsin X co-localization with α- and γ-enolase, protein expression, and activity in enriched and differentiated SH-SY5Y cell populations. **A** Representative images of immunofluorescence staining for cathepsin X (green) and α-enolase (red, *left*) or γ-enolase (red, *right*). Nuclei were counterstained with DAPI (blue). White arrows indicate areas with strong co-localization. White squares represent zoomed-in areas. Scale bars: 10 μm. The graphs below show the Pearson’s correlation coefficient quantifying the linear relationship between the fluorescence intensities of the two channels. Two independent experiments (*N* = 2) were performed. **B** Representative western blots (top) and quantification (bottom) of the expression of different forms of cathepsin X. Protein levels are normalized to GAPDH. Two independent experiments (*N* = 2) were performed. **C** ELISA results for the expression of all forms of cathepsin X. Three independent experiments (*N* = 3) were performed, each in duplicate. **D** Cathepsin X activity based on cathepsin X-specific substrate (Abz-Phe-Glu-Lys(Dnp)-OH). Three independent experiments (*N* = 3) were performed, each in duplicate. Data were obtained after 10 days of enrichment and differentiation and are expressed as means ± SEM (one-way ANOVA, Tukey’s test; * *P* < 0.05)

Based on the increased expression of γ-enolase in N-type cells, we next analyzed the expression and activity of cathepsin X, which has a regulatory effect on the neurotrophic activity of γ-enolase. Confocal microscopy revealed that the co-localization areas of both enolase isoforms and cathepsin X were significantly smaller in S-type cells compared to control and N-type cells, where co-localization was prominently observed at the cell membrane (Fig. 3A). The stronger interaction of enolase isoforms with cathepsin X observed in N-type cells was further confirmed by immunoprecipitation assay (Supplemental Fig. S3). We were further interested in the mature form of cathepsin X, as only this form possesses proteolytic activity. Therefore, we first examined cathepsin X maturation by assessing procathepsin X levels. Western blotting revealed a trend toward increased procathepsin X in S- and N-type cells compared to control cells (Fig. 3B). Conversely, the levels of all forms of cathepsin X did not differ, as also revealed by ELISA assay (Fig. 3B, C). Moreover, enzyme activity assay revealed significantly increased cathepsin X activity in S-type cells (Fig. 3D). These findings indicate that the increase in cathepsin X expression and activity is specific to S-type cells, with no significant differences observed between N-type and non-differentiated controls, suggesting a possible regulatory role of cathepsin X in the neurotrophic activity of γ-enolase.

### Inhibition of Cathepsin X Alters the Expression of the Active γ-Enolase Form in N-Type Cells

**Fig. 4:**
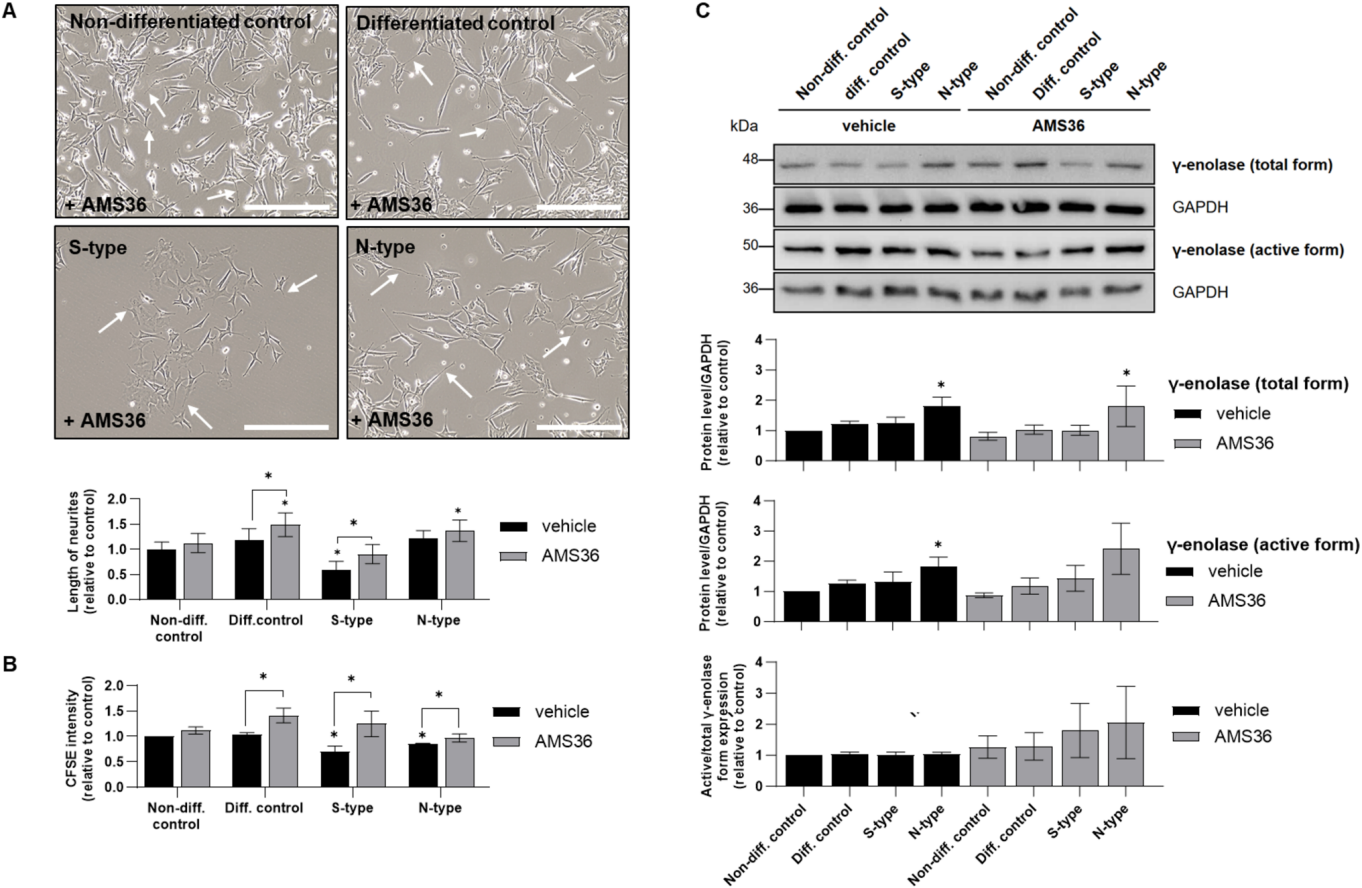
The effects of cathepsin X inhibition on cell morphology, proliferation, and γ-enolase expression in enriched and differentiated SH-SY5Y cell populations. **A** Representative phase-contrast images of SH-SY5Y cell populations after AMS36 treatment. White arrows indicate cell extensions. Scale bars: 100 μm. The neurite lengths (bottom graph) were determined in pixels when the extensions were longer than the cell diameter. The neurite lengths were measured using ImageJ software. Three independent experiments (*N* = 3) were performed, each in duplicate. **B** The proliferation rates of SH-SY5Y cell populations after AMS36 treatment, as assessed with carboxyfluorescein succinimidyl ester (CFSE) and flow cytometry. Three independent experiments (*N* = 3) were performed, each in duplicate. **C** Representative western blots (top) and quantification (bottom) of total and active γ-enolase expressions and their respective ratios after AMS36 treatment. Protein levels are normalized to GAPDH. Four independent experiments (*N* = 4) were performed. Data were obtained after 10 days of enrichment and differentiation and are expressed relative to control and as means ± SEM (one-way ANOVA, Tukey’s test; * *P* < 0.05)

**Fig. 5:**
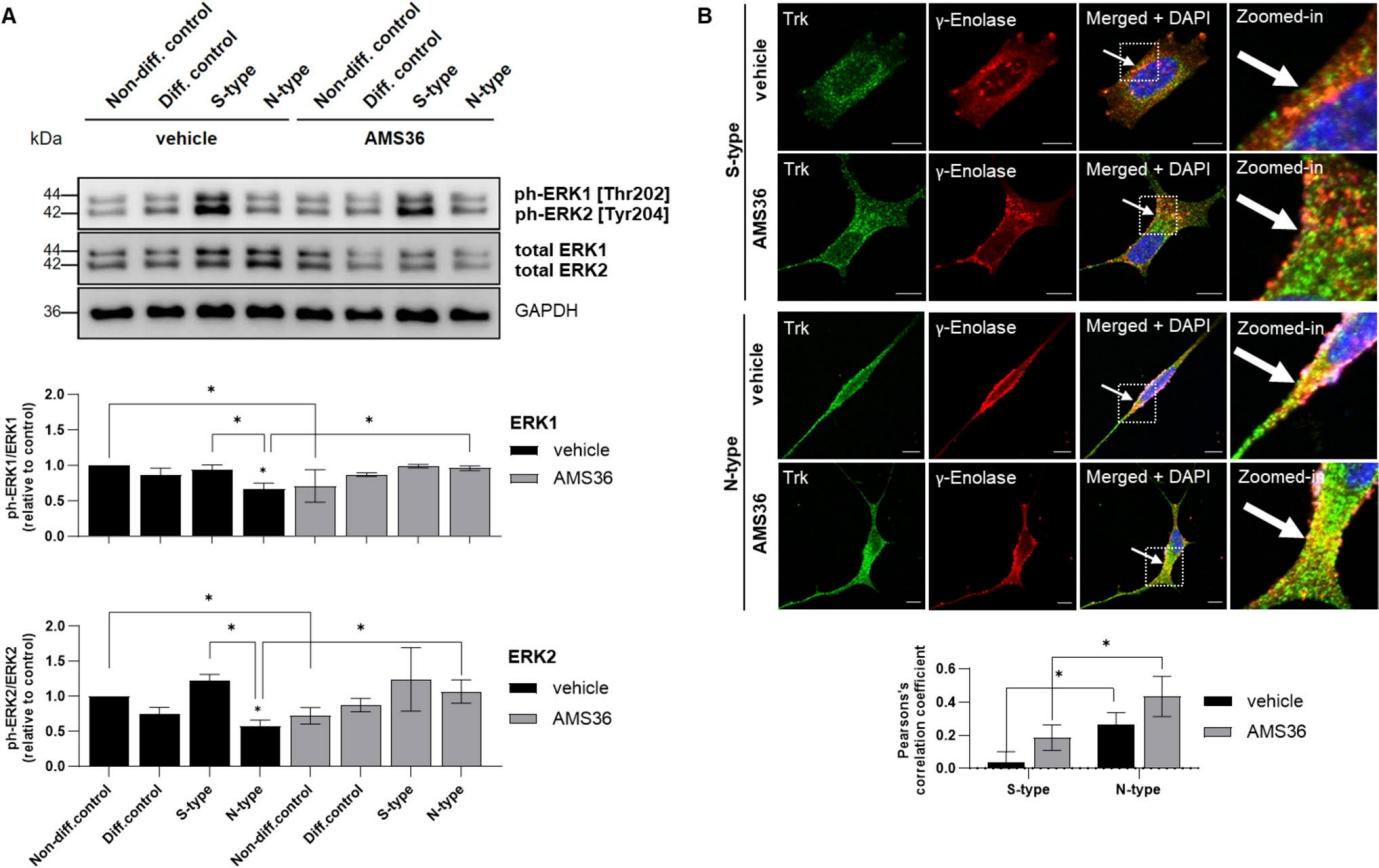
The effects of cathepsin X inhibition on ERK1/2 activation and on association of γ-enolase with the intracellular domain of Trk in SH-SY5Y cell populations. **A** Representative western blots (top) and quantification (bottom) of relative values of the phosphorylated form compared to the total form of ERK1/2 after AMS36 treatment. Protein levels are normalized to GAPDH. **B** Representative images of immunofluorescence staining for Trk (green) and γ-enolase (red, *right*). Nuclei were counterstained with DAPI (blue). White arrows indicate areas with strong co-localization. White squares represent zoomed-in areas. Scale bars: 10 μm. The graph below shows Pearson’s correlation coefficient quantifying the linear relationship between the fluorescence intensities of the two channels. Two independent experiments (*N* = 2) were performed. Data were obtained after 10 days of enrichment and differentiation and are expressed relative to control and as means ± SEM (one-way ANOVA, Tukey’s test; * *P* < 0.05)

To further elucidate the role of cathepsin X and its possible regulation of γ-enolase in S- and N-type cells, we used the cathepsin X inhibitor AMS36. AMS36 induced significant morphological changes, particularly increasing neurite outgrowth, which may suggest enhanced neuronal differentiation (Fig. 4A). AMS36 reduced the proliferation rates in all SH-SY5Y cell phenotypes, with the highest increase in CFSE intensity in S-type cells compared to vehicle-treated cells (Fig. 4B). Western blotting revealed that the ratio between active and total γ-enolase remained unchanged in S- and N-type cells exposed to vehicle (Fig. 4C). Conversely, AMS36 appeared to increase the ratio of active:total γ-enolase forms in both S- and N-type cells (Fig. 4C), although this increase was not statistically significant. Additionally, we determined the effects of AMS36 on the levels of characteristic markers of S- and N-type cells. AMS36 decreased vimentin levels in S- and N-type cells and Bcl-2 levels in differentiated cells and S- and N-type cells; however, these decreases were not statistically significant (Supplemental Fig. S4). We further investigated the impact of AMS36 on the activation of MAPK signaling pathway, which is involved in cell proliferation and differentiation processes. In vehicle-treated cells, ERK1/2 phosphorylation was increased in S-type cells compared to N-type cells and decreased in N-type cells compared to the control (Fig. 5A). AMS36 treatment did not affect ERK1/2 activation in S-type cells, however, ERK1/2 phosphorylation significantly differed between AMS36-treated and vehicle-treated N-type cells (Fig. 5A). This correlates with increased expression of the active γ-enolase form in N-type cells in presence to AMS36 compared to inhibitor non-treated cells. Therefore, the association of γ-enolase with the tyrosine receptor kinase (Trk) in AMS36-treated cells was observed (Fig. 5B). The treatment with AMS36 altered the co-localization rate between Trk and γ-enolase. Specifically, the co-localization rate was higher in N-type cells compared to S-type cells. In S-type cells, AMS36 treatment increased the co-localization rate relative to the vehicle-treated cells. Moreover, AMS36-treated N-type cells exhibited the highest co-localization rate between Trk and γ-enolase, though this increase was not significant when compared to vehicle-treated N-type cells. The enhanced co-localization observed with AMS36 treatment might have potential implications for Trk signaling, which is critical for neuronal survival and differentiation triggered through activated MAPK signaling pathway. Our findings suggest a regulatory role of cathepsin X in influencing γ-enolase activity, as indicated by its membrane localization and impact on signaling pathways. We indirectly showed that cathepsin X inhibition increases the active form of γ-enolase, particularly in N-type cells, which may contribute to its neurotrophic activity and role in neuronal-like cells.

## Discussion

Our study used the SH-SY5Y cell line to assess α- and γ-enolase expression in S- and N-type cells and the role of γ-enolase in relation to cathepsin X. We observed that γ-enolase levels were higher in N-type cells compared to other cell types, where its expression was also detected. Additionally, α-enolase was ubiquitously expressed in all cell types. Moreover, we found that cathepsin X regulates neuronal enhancement and differentiation by modulating the expression of γ-enolase and phenotype-specific markers, with distinct proteolytic activity in S-type cells and ERK1/2 activation in AMS36-treated N-type cells.

We adapted a protocol previously established by Bell et al. [6] for the enrichment and differentiation of SH-SY5Y cells into S- and N-type cells. For 10 days, we observed significant phenotypic and molecular distinctions between the two cell types. S-type cells were identified by their large, flat cell bodies and strong substrate adherence, indicative of an epithelial-like phenotype. N-type cells displayed smaller, slightly elongated bodies and branched, neurite-like extensions, indicative of differentiation towards a neuronal lineage [6]. Our observations are consistent with the literature describing the morphological criteria for neuronal differentiation, reinforcing the utility of SH-SY5Y cells as a model for studying neurodevelopmental and neurodegenerative diseases [6, 7, 18, 19]. Additionally, CFSE fluorescence intensity was decreased in S-type cells, indicating increased cell proliferation and arrested differentiation [20]. These results align with a previous study demonstrating that short (5-day) treatment with retinoic acid induces N-type cell differentiation, whereas long (10-day) treatment promotes S-type cell proliferation [19].

The specific molecular markers identified through western blotting further confirmed the phenotypic characterization of S- and N-type cells. Vimentin, an intermediate filament protein typically found in non-neuronal cells [3], was increased in S-type cells, reflecting their epithelial-like morphology. Neurofilament light chain protein, a cytoskeletal component in mature neurons [1], was increased in N-type cells. Tyrosine hydroxylase is another marker with higher expression in N-type cells and relatively low expression in S-type cells [21]. GAP-43, a marker of axon growth, was increased in N-type cells, even though previous reports did not demonstrate different expression between the phenotypes [18]. The anti-apoptotic protein Bcl-2 was increased in N-type cells, aligning obtained results with previous studies [6, 7].

Our study further investigated the differential expression of enolase isoforms and functional implications of γ-enolase in S- and N-type cells, providing new insights into the role of enolase in cell differentiation. It is known that α-enolase is ubiquitously expressed, whereas γ-enolase is specifically expressed. In this study, α-enolase was expressed in all SH-SY5Y cell populations with no differences among phenotypes, reflecting its role as a housekeeping enzyme essential for metabolism across neuronal and non-neuronal cells [8]. However, γ-enolase was more highly expressed in N-type cells but was also detectable in S-type and undifferentiated cells, as confirmed by western blotting, ELISA, and confocal immunofluorescence microscopy, suggesting that γ-enolase could be used as a marker for N-type cells. Moreover, our findings reveal a dynamic shift in the expression profiles of α- and γ-enolase isoforms during the enrichment process of SH-SY5Y cells. As the enrichment process progressed, an evident transition occurred during which γ-enolase levels began to increase and eventually surpassed those of α-enolase by day 10, particularly in N-type cells. This increased γ-enolase expression in N-type cells aligns with their differentiation towards a more neuron-like phenotype, characterized by the extension of neurites and expression of neuronal markers. As such, α- and γ-enolase expression profiles and their shift over time could serve as markers of cell maturity and differentiation state. This is further supported by our time-based profiling of other neuronal markers, such as GAP-43 and Bcl-2, known for their roles in neurite outgrowth and cell survival, respectively [22, 23]. The differential expressions of these markers at different time points support the concept that the cell populations undergo phenotypic changes, which coincide with shifting enolase isoform profiles.

In this study, glycolytic activity measured by the enolase activity assay did not differ among phenotypes, suggesting that it is not affected by γ-enolase upregulation. As reported by Takei et al. [24], the correlation between the glycolytic activity and the neurotrophic effect of γ-enolase appears to be minimal. Low concentrations of γ-enolase can elicit a neurotrophic response, whereas the concentrations of enzymatic reaction substrate (2-phosphoglycerate) and product (phosphoenolpyruvate) have no effect [24].

As γ-enolase levels were increased in N-type SH-SY5Y cells, our research focused primarily on γ-enolase due to its well-established neuritogenic effect mediated by its C-terminal part. Conversely, C-terminal end of α-enolase predominantly serves as a plasminogen receptor, a role less relevant to the neuritogenic activity [8]. Additionally, previous studies of direct interactions between γ-enolase and cathepsin X provided further support, particularly given reports of stronger interaction at the plasma membrane of BV2 microglial cells after lipopolysaccharide stimulation [25] and in differentiated neuronal PC12 cells, in which cathepsin X and γ-enolase co-localize predominantly at neurite growth cones [15]. Moreover, strong co-localization of cathepsin X and γ-enolase was observed in surrounding of beta-amyloid plaques in a mouse model of Alzheimer’s disease [14]. Our current study revealed notable co-localization of α- or γ-enolase and cathepsin X at the cell membrane in N-type cells, but only weak co-localization in S-type cells. This pattern suggests that interactions between α- or γ-enolase and cathepsin X are only relevant in N-type cells, i.e., neuron-like or differentiated neuronal cells. Immunoprecipitation further validated this interaction. Furthermore, our results showed distinct expression patterns of cathepsin X, and since our focus was on its active, proteolytic form, we performed enzyme activity assays, which confirmed increased activity in S-type cells. These phenotype-specific variations raise questions about the regulatory mechanisms controlling cathepsin X levels and activity in different cell types.

To assess the role of cathepsin X in regulating γ-enolase expression in S- and N-type cells, we used the cathepsin X inhibitor AMS36 at a 10 μM concentration. This concentration was chosen based on previous studies demonstrating its efficacy and biological relevance. In Pišlar et al. (2014), 10 μM AMS36 significantly inhibited cathepsin X activity, reducing 6-hydroxydopamine-induced apoptosis in PC12 and SH-SY5Y neuronal cells while maintaining minimal cytotoxicity. Similarly, Pišlar et al. (2017) showed that 10 μM AMS36 suppressed cathepsin X activity in microglial cells, leading to decreased microglial activation and inflammatory cytokine production. These studies support the use of 10 μM AMS36 as an effective and safe inhibitor for investigating cathepsin X-mediated regulation of γ-enolase. The cathepsin X inhibitor AMS36 caused significant morphological changes, particularly enhancing neurite outgrowth in S-type cells. It also decreased cell proliferation and active γ-enolase levels in both S- and N-type cells. Furthermore, AMS36 slightly decreased vimentin expression and significantly decreased Bcl-2 expression, reflecting changes in cellular structure and function. Their decreased expression may be associated with cell differentiation, as previous studies reported increased neuronal differentiation in the absence of vimentin phosphorylation [26] and decreased Bcl-2 expression during the differentiation of adipose-derived stromal cells into neurons [27].

In addition to increasing active γ-enolase levels, AMS36 activated ERK1/2 in N-type cells, which correlates with the role of γ-enolase in activating MAPK pathways. This indicates the crucial role of γ-enolase in cell survival and neurite outgrowth, which has also been demonstrated in previous studies. The γ-enolase C-terminal peptide promotes cell survival and neurite outgrowth via phosphatidylinositol-3-kinase/Akt and MAPK/ERK pathways, and these effects depend on Trk kinase activity, as revealed by inhibiting Trk kinase activity with K252a [12, 28]. Therefore, the association of γ-enolase with the Trk receptor in both SH-SY5Y cell types would reveal the role of upregulated γ-enolase in N-type cells. Nevertheless, ERK1/2 activation is typically associated with proliferation and differentiation, and its predominant activity observed in S-type cells might promote proliferation and inhibit neuronal differentiation, as observed in neural stem cells [29]. The complexity of ERK1/2 activation in distinct SH-SY5Y cell phenotypes warrants further investigations to elucidate their roles in neuronal differentiation, proliferation, and survival. Our findings revealed an increase in the co-localization between Trk and γ-enolase upon AMS36 treatment, particularly in N-type cells compared to S-type cells. This enhanced co-localization aligns with the mechanism proposed by Pišlar and Kos, where γ-enolase promotes Trk receptor internalization and trafficking to late endosomes, essential for Rap1 activation and subsequent neurite outgrowth. Inhibiting cathepsin X, thereby preventing the cleavage of γ-enolase, likely enhances this co-localization and interaction with Trk, resulting in similar neurotrophic effects. While the enhanced co-localization observed with AMS36 treatment might suggest functional implications for Trk signaling, the differences observed in our study warrant further investigation.

## Conclusion

This study provides insights into the roles of enolase isoforms and cathepsin X in neuronal differentiation and phenotype-specific behaviors within the SH-SY5Y cell model. We demonstrate that γ-enolase, in addition to its known role as a neurotrophic factor for neuronal survival and neurite outgrowth, plays a role in neuronal differentiation. Our results reveal a switch in expression from α-enolase to γ-enolase during enrichment and differentiation towards S- and N-type SH-SY5Y cells, with increased γ-enolase expression in enriched N-type cells, indicating its function in differentiated neuron-like cells. Moreover, the proteolytic activity of cathepsin X, a regulator of γ-enolase neurotrophic activity, was increased in S-type cells. Additionally, inhibiting cathepsin X caused longer cell extensions and higher differentiation rates in all cell phenotypes. Inhibiting cathepsin X also increased γ-enolase expression and possibly promoted differentiation by downregulating vimentin and Bcl-2 levels and influenced the signaling pathways involved in this process. Future investigations should focus on understanding these regulatory mechanisms, which would improve our understanding of neuronal development. The SH-SY5Y cell line, with its distinct phenotypes, serves as an appropriate model for studying neuronal differentiation. Our findings provide a foundation for future studies on the differentiation of SH-SY5Y cells into specific neuronal subtypes, such as dopaminergic, adrenergic, and cholinergic neurons. Furthermore, the observed roles of γ-enolase and cathepsin X in neuronal differentiation suggest potential therapeutic relevance for neurodegenerative diseases, where γ-enolase could support neuronal survival and differentiation, and cathepsin X inhibition may enhance these processes. Future research should aim to clarify these mechanisms in more physiologically relevant models to better understand their translational potential towards therapeutic development.

## Supplementary Information

Below is the link to the electronic supplementary material.

ESM 1 (DOCX 3.65 MB)

ESM 2 (DOCX 2.38 MB)

## Data Availability

No datasets were generated or analysed during the current study.

## Abbreviations

Bcl-2: B-cell lymphoma 2
CFSE: Carboxyfluorescein succinimidyl ester
ERK: Extracellular signal-regulated kinase
GAP-43: Growth-associated protein-43
MAPK: Mitogen-activated protein kinase
SEM: Standard error of the mean
Trk: Tyrosine receptor kinase
