# Supplementary material for: α-Enolase and γ-Enolase Expression in Enriched S- and N-Type SH-SY5Y Cells: Regulatory Role of Cathepsin X
Source: Mol Neurobiol. 2025 Apr 3;62(8):10006–19. doi: 10.1007/s12035-025-04898-2 (PMC12289780; doi:10.1007/s12035-025-04898-2)
Supplement: Supplementary file 1 — (DOCX 3.65 MB) [file 12035_2025_4898_MOESM1_ESM.docx]

**Supplementary**

**A
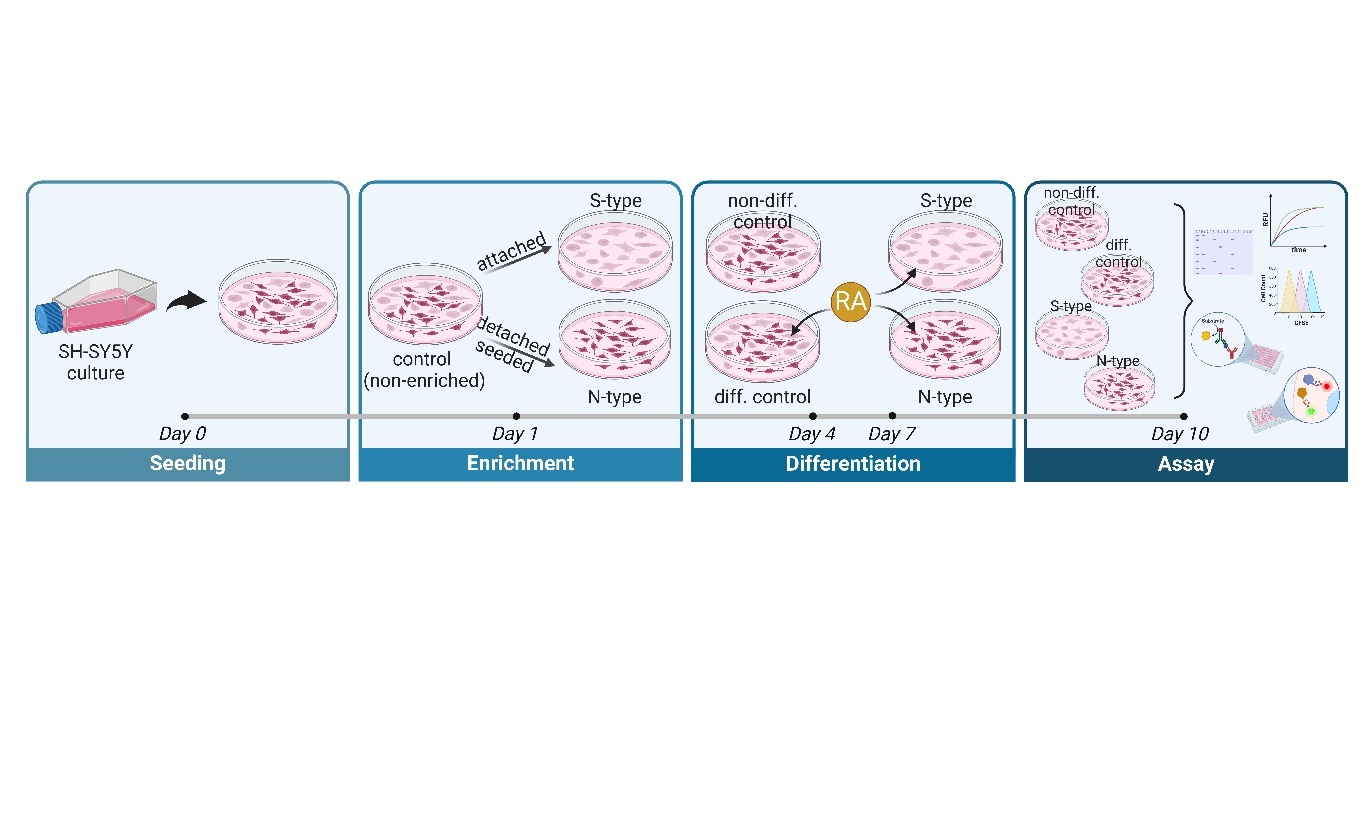
**

**B**

**
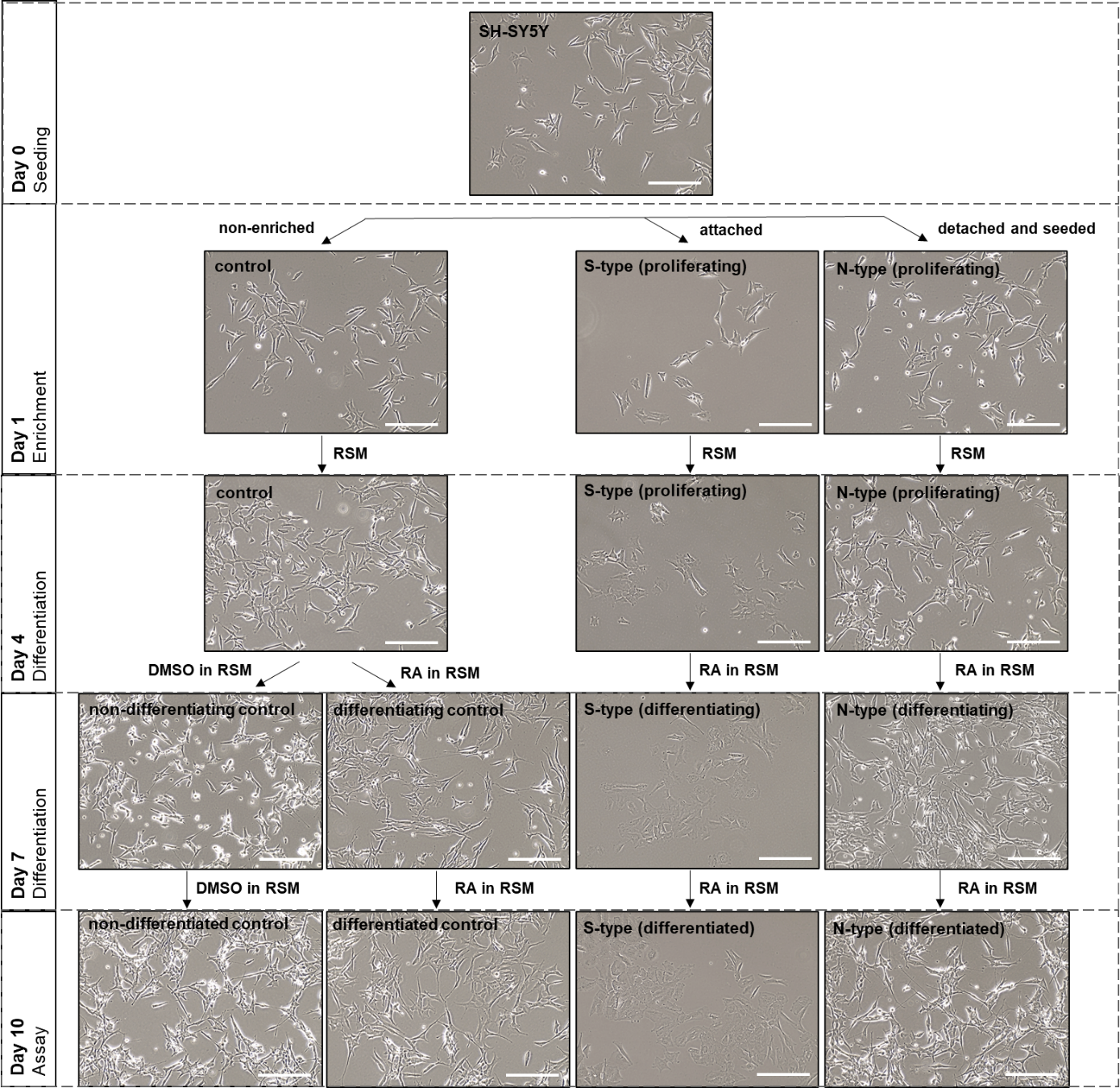
Fig. S1. Enrichment and differentiation of S- and N-type SH-SY5Y cell populations****.** SH-SY5Y cells cultured in complete media were washed with phosphate-buffered saline (PBS). Detached cells were transferred to a new culture plate with complete media (N-type cells), and the remaining adhered cells were cultured in reduced serum media (RSM) (S-type and control cells). After 4 and 7 days, retinoic acid (RA, 5 μM) in RSM was added to facilitate cell enrichment during the differentiation process for a total of 10 days. (**A**) Schematic flowchart of the enrichment and differentiation protocol. (**B**) Representative phase-contrast images of SH-SY5Y cell populations on days 0, 1, 4, 7, and 10 of the enrichment and differentiation protocol. Scale bars: 100 μm.


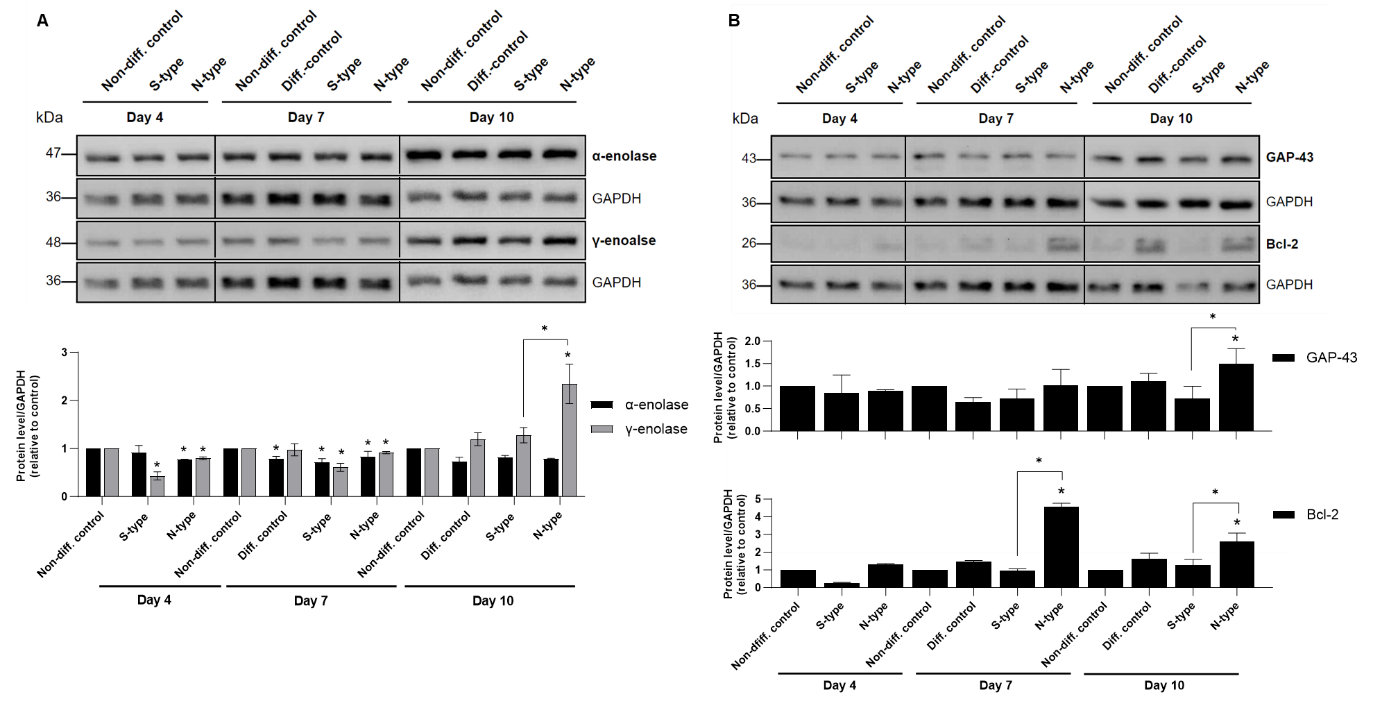


**Fig. S2. Protein expressions of α-enolase, γ-enolase, and specific markers during enrichment and differentiation of SH-SY5Y cell populations.** (**A**) Representative western blots (top) and quantification (bottom) of α-enolase and γ-enolase expressions. Three independent experiments (N=3) were performed. (**B**) Representative western blots (top) and quantification (bottom) of GAP-43 and Bcl-2 expressions. Two independent experiments (N=2) were performed. Protein levels are normalized to GAPDH. Data were obtained after 4, 7 and 10 days of enrichment and differentiation and are expressed relative to control and as means ± SEM (one-way ANOVA, Tukey’s test, * P < 0.05).

**
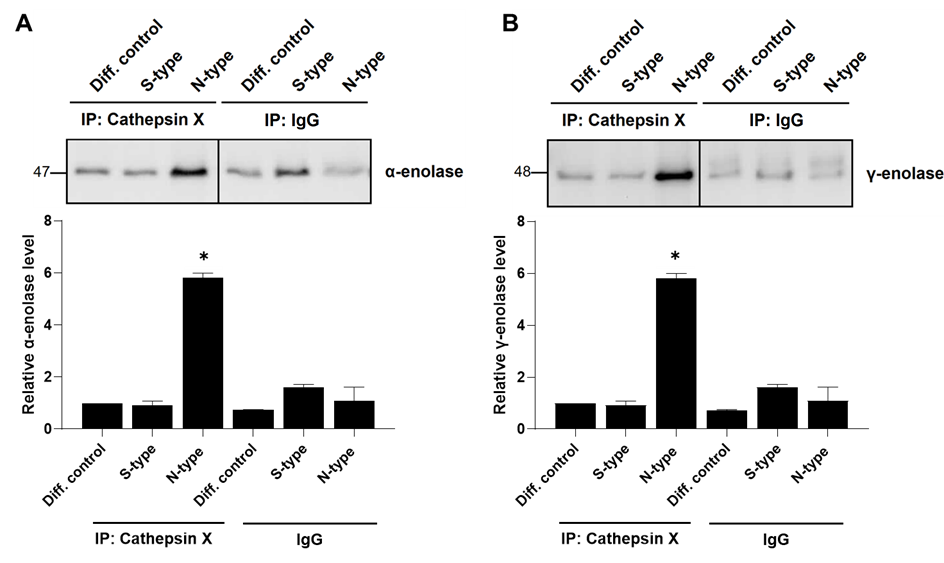
**

**Fig. S3. Immunoprecipitation of cathepsin X with α-enolase and γ-enolase in SH-SY5Y cell populations.** (**A, B**) Representative western blots (top) and quantification (bottom) of α-enolase (**A**) and γ-enolase (**B**) expressions after immunoprecipitation with cathepsin X. Two independent experiments (N=2) were performed. Data were obtained after 10 days of enrichment and differentiation and are expressed as relative to control and as means ± SEM (one-way ANOVA, Tukey’s test, * P < 0.05).


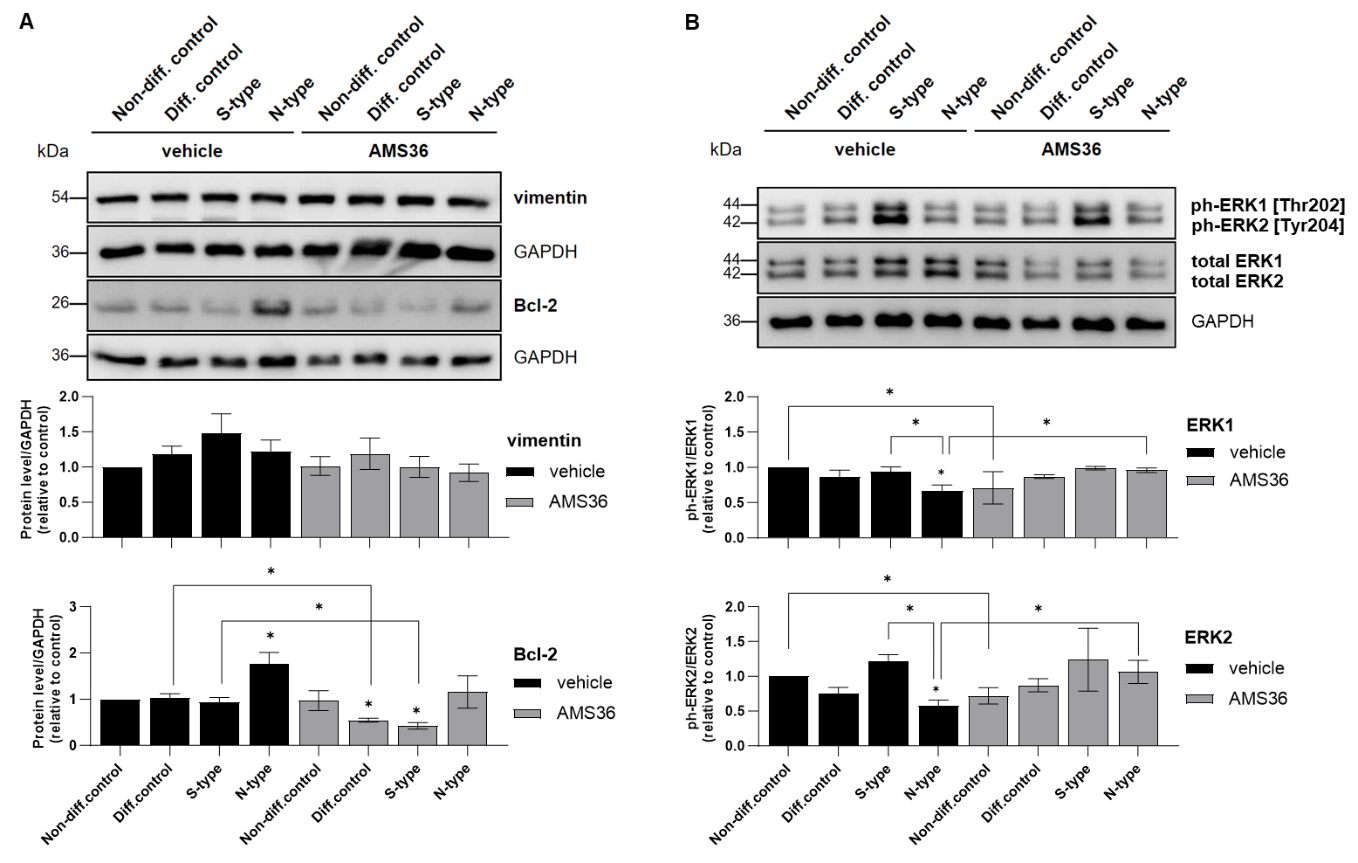


**Fig. S4. The effects of cathepsin X inhibition on the expression of specific markers in SH-SY5Y cell populations.** Representative western blots (top) and quantification (bottom) of vimentin and Bcl-2 expressions after AMS36 treatment. Protein levels are normalized to GAPDH. Data were obtained after 10 days of enrichment and differentiation and are expressed relative to control and as means ± SEM (one-way ANOVA, Tukey’s test; * P < 0.05).
