# Supplementary material for: α-Enolase and γ-Enolase Expression in Enriched S- and N-Type SH-SY5Y Cells: Regulatory Role of Cathepsin X
Source: Mol Neurobiol. 2025 Apr 3;62(8):10006–19. doi: 10.1007/s12035-025-04898-2 (PMC12289780; doi:10.1007/s12035-025-04898-2)
Supplement: Supplementary file 2 — (DOCX 2.38 MB) [file 12035_2025_4898_MOESM2_ESM.docx]

**Supplementary Material - Raw images of western-blotted membranes**

**
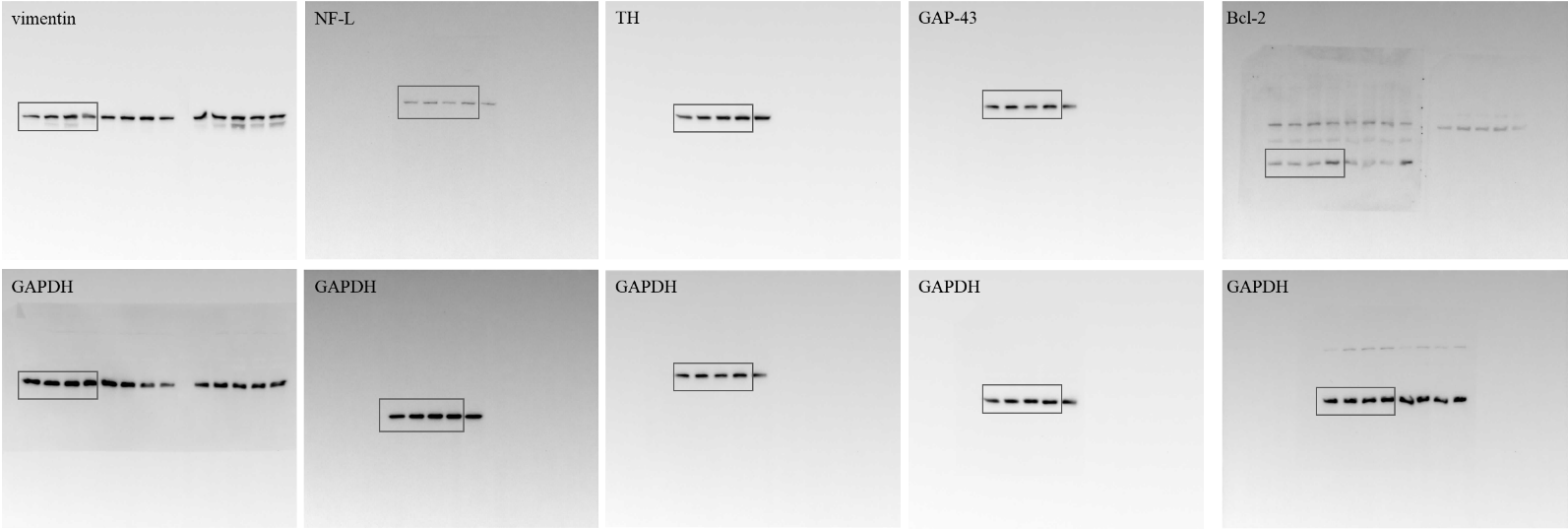
**

**Addition to Fig. 1C:** Raw images of the representative western-blotted membranes of the expression of vimentin, NF-L, TH, GAP-43 and Bcl-2 with the appropriate representative western-blotted membranes of the expression of GAPDH.


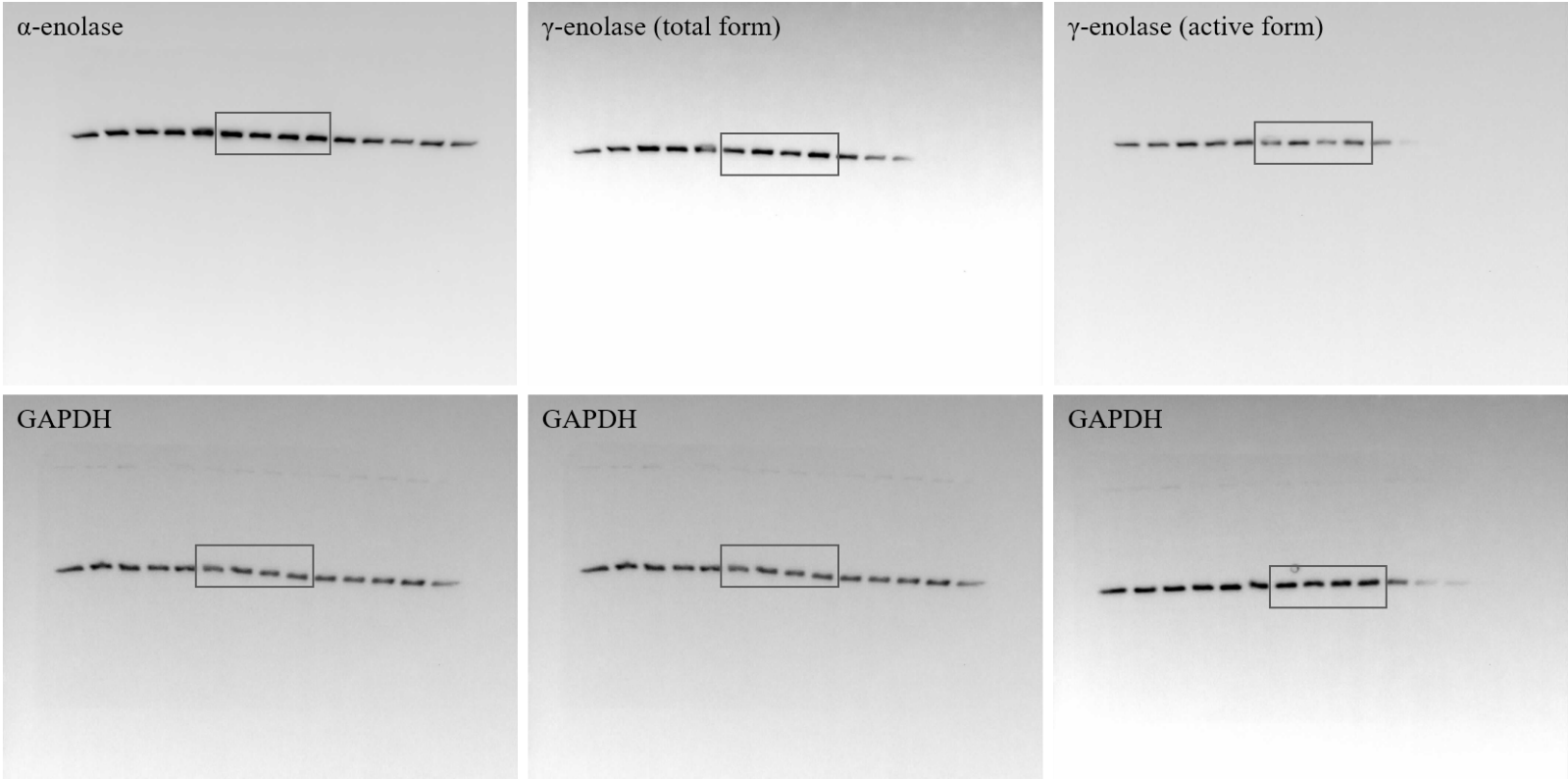


**Addition to Fig. 2A:** Raw images of the representative western-blotted membranes of the expression of α-enolase, total form of γ-enolase and active form of γ-enolase with the appropriate representative western-blotted membranes of the expression of GAPDH.


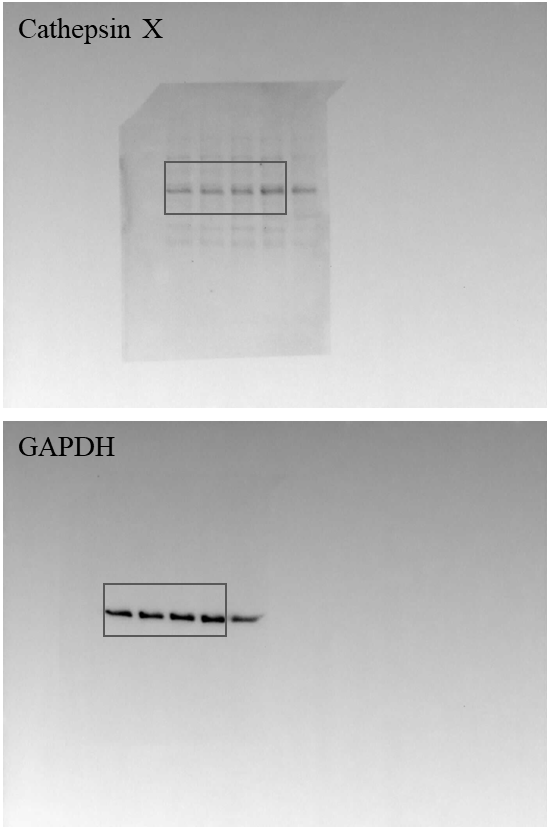


**Addition to Fig. 3B:** Raw image of the representative western-blotted membrane of the expression of cathepsin X with the appropriate representative western-blotted membrane of the expression of GAPDH.

**
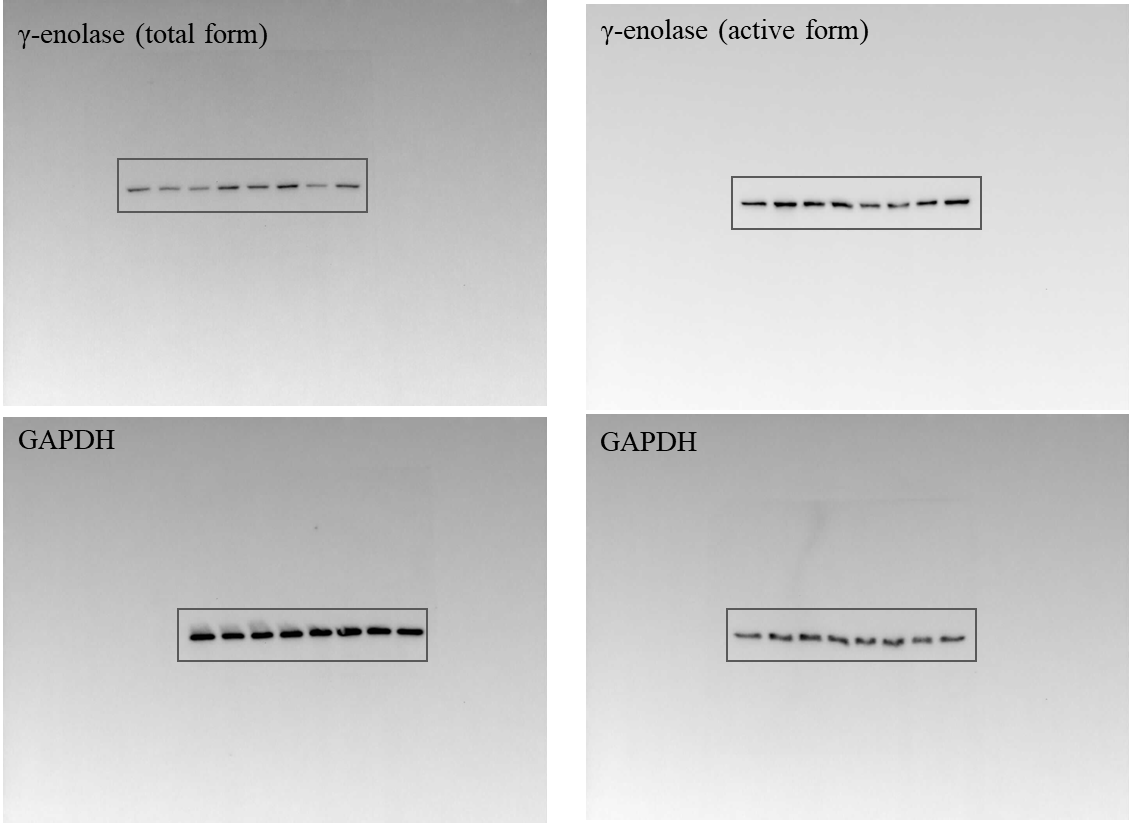
**

**Addition to Fig. 4C:** Raw images of the representative western-blotted membranes of the expression of total form of γ-enolase and active form of γ-enolase with the appropriate representative western-blotted membranes of the expression of GAPDH.

**
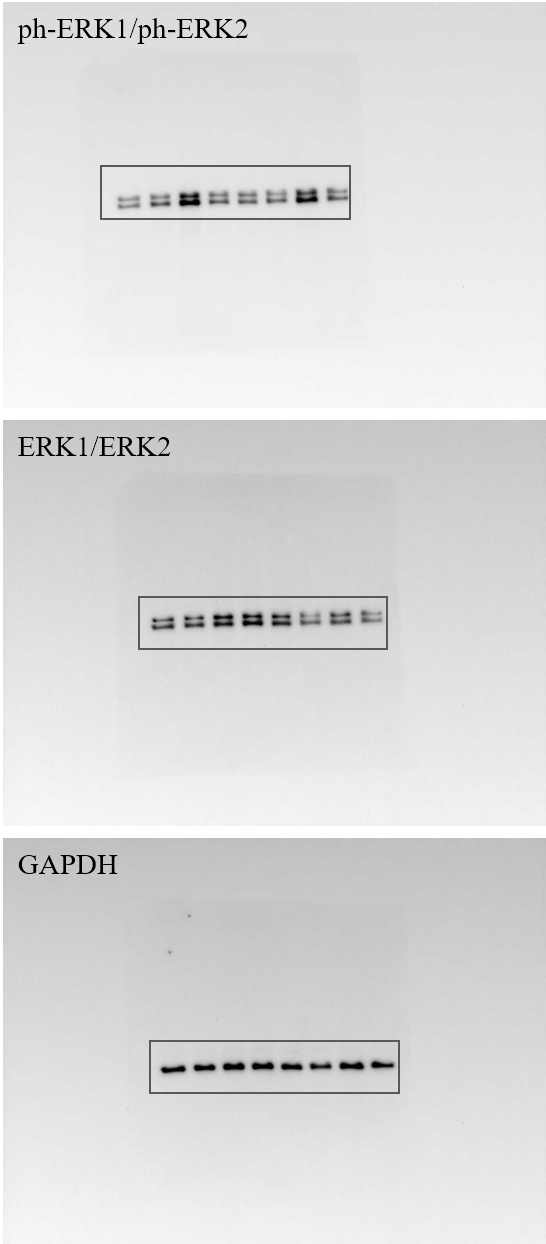
**

**Addition to Fig. 5A:** Raw images of the representative western-blotted membranes of the expression of phosphorylated ERK1/2 and total ERK1/2 with the appropriate representative western-blotted membranes of the expression of GAPDH.


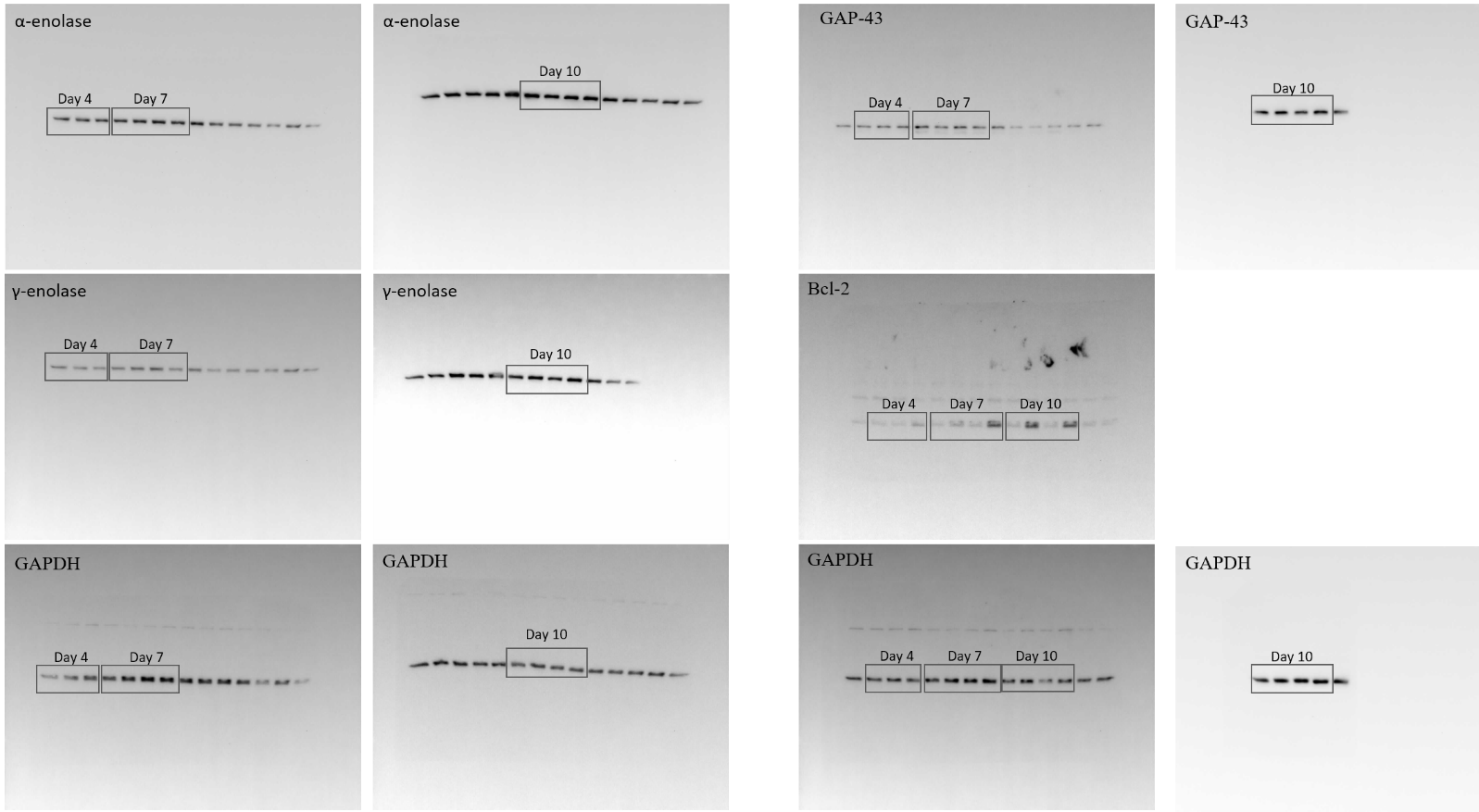


**Addition to Fig. S2:** Raw images of the representative western-blotted membranes of the expression of α-enolase and γ-enolase (Fig. S2A) and GAP-43 and Bcl-2 (Fig. S2B) with the appropriate representative western-blotted membranes of the expression of GAPDH.


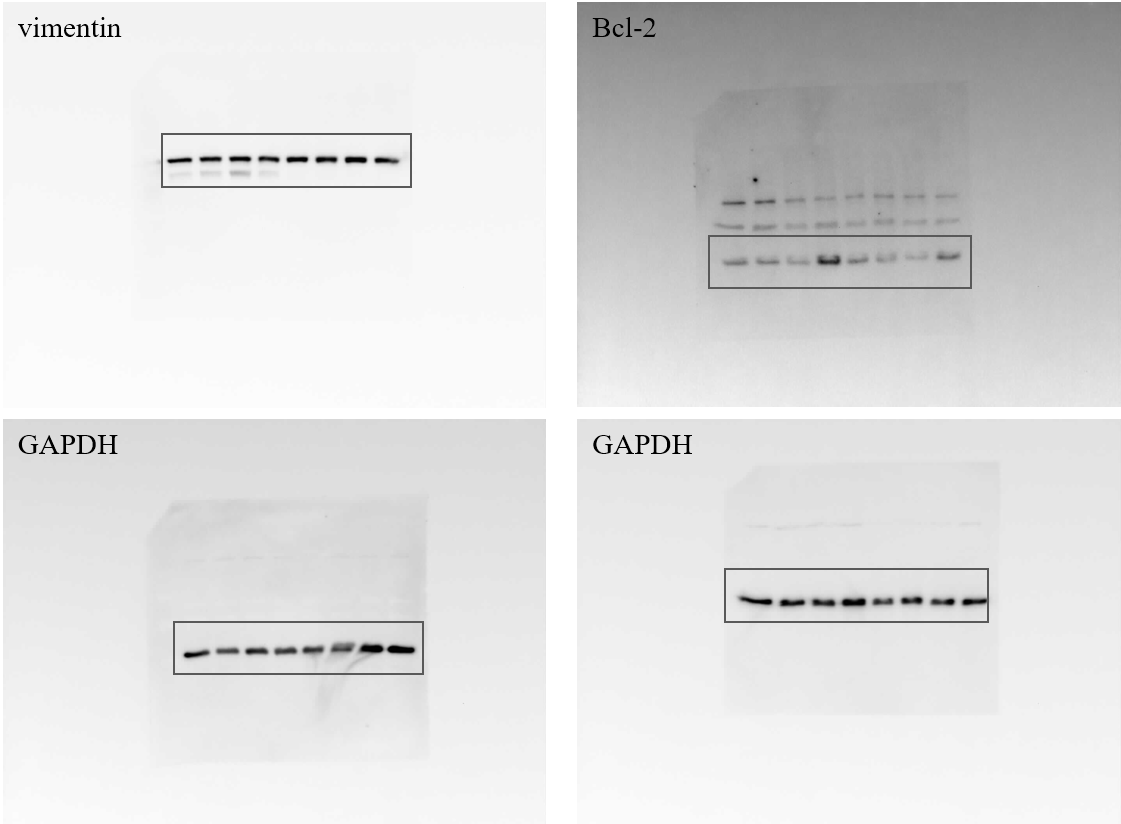


**Addition to Fig. S3:** Raw images of the representative western-blotted membranes of the expression of GAP-43 and Bcl-2 with the appropriate representative western-blotted membranes of the expression of GAPDH.
